# Contribution of cytochrome P450-mediated metabolism to clavulanic acid-induced cytotoxicity in TK6-derived cytochrome P450-overexpressing cell lines

**DOI:** 10.1038/s41598-026-59728-9

**Published:** 2026-07-15

**Authors:** Jia-Long Fang, Xilin Li, Si Chen, Frederick A. Beland

**Affiliations:** 1 Division of Biochemical Toxicology, National Center for Toxicological Research (NCTR), U.S. Food and Drug Administration, 3900 NCTR Road, Jefferson, AR 72079 USA; 2 Division of Genetic and Molecular Toxicology, National Center for Toxicological Research (NCTR), U.S. Food and Drug Administration, 3900 NCTR Road, Jefferson, AR 72079 USA

**Keywords:** Clavulanic acid, Cytotoxicity, Cytochrome P450, Apoptosis, DNA damage, Biochemistry, Diseases, Drug discovery, Medical research

## Abstract

Clavulanic acid, a β-lactamase inhibitor, is widely co-administered with amoxicillin (as in *Augmentin*) to treat a broad range of mild-to-moderate bacterial infections by inhibiting β-lactamase enzymes produced by resistant bacterial strains. Despite its therapeutic efficacy, clavulanic acid has been associated with drug-induced liver injury, but the underlying mechanisms for this toxicity remain poorly understood. We investigated the contribution of cytochrome P450 (CYP)-mediated metabolism in relation to clavulanic acid-induced cytotoxicity. A panel of TK6-derived cell lines overexpressing 18 individual CYP isoforms (CYP1A1, CYP1A2, CYP1B1, CYP2A6, CYP2A7, CYP2A13, CYP2B6, CYP2C8, CYP2C9, CYP2C18, CYP2C19, CYP2D6, CYP2E1, CYP3A4, CYP3A5, CYP3A7, CYP4A11, and CYP4B1) was employed. Cells were incubated for 24 and 48 h with freshly prepared clavulanic acid (20-1000 µM) in RPMI-1640 medium, and the half-maximal inhibitory concentration (IC₅₀) was determined. Cell growth was more markedly delayed after 48 h of treatment than after 24 h of exposure. Following 48 h of treatment, 13 CYP-overexpressing cell lines showed reduced IC₅₀ values compared with parental TK6 and TK6/vector control cells. The rank order was CYP3A4 ≈ CYP3A7 ≈ CYP1A1 < CYP4A11 ≈ CYP2A13 ≈ CYP2C19 < CYP2B6 ≈ CYP1A2 ≈ CYP2C9 < CYP1B1 ≈ CYP2D6 < CYP2E1 ≈ CYP2C18, with the greatest decrease (~2-fold) observed in TK6/CYP3A4 cells. In contrast, five isoforms (CYP2A6, CYP2A7, CYP2C8, CYP3A5, and CYP4B1) showed IC₅₀ values comparable to controls. To examine further the cytotoxic response, TK6/CYP vector and TK6/CYP3A4 cells were treated for 48 h with equitoxic concentrations of clavulanic acid (0.1×, 0.5×, and 1.0× IC₅₀). The exposures resulted in a significant concentration-dependent reduction in cell viability, accompanied by increased lactate dehydrogenase release, the induction of apoptosis, G₀/G₁ phase arrest, and a concentration-dependent elevation in γH2AX phosphorylation, indicative of DNA damage. In conclusion, CYP enzymes, especially CYP3A4, likely contribute to clavulanate-induced cytotoxicity, with necrosis, cell cycle disruption, and γH2AX-associated apoptosis as key cellular responses.

**Supplementary Information:**

The online version contains supplementary material available at https://doi.org/10.1038/s41598-026-59728-9.

## Introduction

**Fig. 1:**
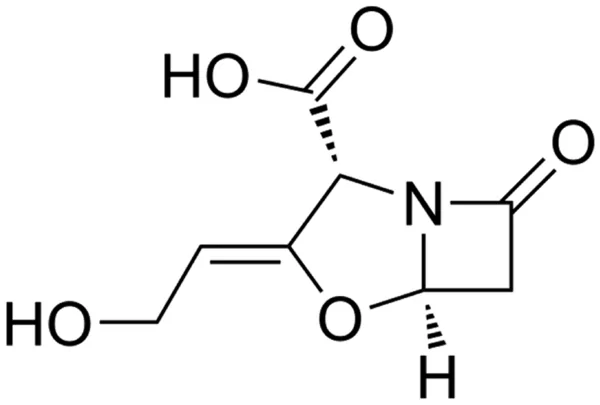
The chemical structure of clavulanic acid.

Clavulanic acid (Fig. 1), a β-lactamase inhibitor, is commonly used in combination with amoxicillin (as in *Augmentin*) to treat a variety of mild-to-moderate bacterial infections. By inhibiting β-lactamase enzymes produced by resistant bacteria, clavulanic acid prevents the enzymatic degradation of amoxicillin and thereby enhances its antibacterial efficacy against β-lactamase-producing pathogens^1,2^. Despite its clinical effectiveness, the amoxicillin-clavulanic acid combination has been associated with a significant risk of drug-induced liver injury (DILI) in both the United States and Europe^3–7^. Notably, accumulating evidence suggests that clavulanic acid, rather than amoxicillin, is a primary contributor to this hepatotoxicity, raising concerns about the safety of its widespread use^6,8^.

Clavulanic acid-associated DILI typically presents with elevations in serum liver enzymes, including alanine aminotransferase and aspartate aminotransferase, and is frequently accompanied by cholestatic features. In many cases, liver injury may be prolonged, with risk potentially influenced by age and sex ^6^. Although rare, severe cases of DILI can progress to acute liver failure and may be fatal^6,9^.

Several host-related risk factors for clavulanic acid-induced hepatic injury have been proposed, including sex-related differences, genetic polymorphisms, and cytochrome P450 (CYP) enzyme activity^10–15^. Sex-based differences in DILI susceptibility have been reported, with some studies indicating a higher risk in women, potentially due to differences in drug metabolism or immune responses^12^. In addition, genetic polymorphisms, particularly in genes encoding drug-metabolizing enzymes or immune-related molecules such as human leukocyte antigen variants, have been associated with increased susceptibility to clavulanic acid-induced liver injury^13,14^.

CYP enzymes are central mediators of xenobiotic metabolism and play a critical role in drug clearance, bioactivation, and the formation of chemically reactive intermediates. CYP-mediated bioactivation is a well-established initiating event in many forms of idiosyncratic DILI, as reactive metabolites can covalently modify cellular macromolecules, induce oxidative stress, disrupt mitochondrial function, and trigger immune-mediated hepatotoxicity^14,16^. Although the specific role of CYP enzymes in clavulanic acid metabolism remains poorly defined, emerging evidence suggests that CYP-mediated metabolism may nonetheless produce reactive intermediates capable of initiating cellular stress responses, particularly in susceptible individuals^17^. Differential involvement of specific CYP isoforms could contribute to variability in cytotoxic outcomes, providing a mechanistic basis for the idiosyncratic nature of clavulanic acid-induced liver injury.

The extent to which CYP enzymes contribute to clavulanic acid metabolism and cytotoxicity remains poorly characterized. To address this knowledge gap, the present study investigated the impact of CYP-mediated metabolism of clavulanic acid on cytotoxicity using TK6-derived cell lines engineered to overexpress individual human CYP isoforms. Cell cycle alterations, apoptosis, and DNA damage were evaluated to elucidate the mechanisms underlying clavulanic acid-induced cytotoxicity.

## Materials and methods

### Chemicals and reagents

Clavulanic acid potassium (CAS #: 61177-45-5; purity: 96%) was obtained from LGC Standards (Toronto, ON, Canada). Bromodeoxyuridine (BrdU), propidium iodide, sodium pyruvate, 2,2-*bis*(hydroxymethyl)-2,2′,2″-nitrilotriethanol (Bis-Tris), and non-essential amino acids were obtained from MilliporeSigma (Burlington, MA). Dulbecco’s modified Eagle medium (DMEM), L-glutamine, penicillin-streptomycin solution, blasticidin, 2.5% trypsin, and RIPA buffer were purchased from Thermo Fisher Scientific, Inc. (Pittsburgh, PA). Fetal bovine serum (FBS) was acquired from Atlanta Biologicals (Lawrenceville, GA). RPMI 1640 medium was purchased from ATCC (Manassas, Virginia).

### Antibodies

Goat polyclonal antibody to human CYP2D6 and mouse monoclonal antibodies to human CYP1A2, CYP3A4, and β-actin were acquired from Santa Cruz Biotechnology (Santa Cruz, CA). Rabbit polyclonal antibody to human CYP4A11 was obtained from Thermo Fisher Scientific, Inc. Rabbit polyclonal antibodies to human CYP1A1, CYP2A6, CYP2A13, CYP2C18, and CYP4B1, rabbit monoclonal antibodies to human CYP1B1, CYP2B6, CYP2C9, CYP2C19, CYP2E1, CYP3A5, and CYP3A7, mouse polyclonal antibody to human CYP2A7, and goat polyclonal antibody to human CYP2C8 were obtained from Abcam Inc. (Cambridge, MA). Rabbit polyclonal antibody to phosphorylated histone H2AX at Ser139 (γH2AX) was obtained from Cell Signaling Technology (Danvers, MA). The fluorescein isothiocyanate (FITC)-conjugated monoclonal anti-BrdU antibody (clone B44) was purchased from BD Biosciences (San Jose, CA).

### Cell culture and chemical treatments

Human lymphoblast TK6 cells were obtained from ATCC and cultured in RPMI 1640 medium supplemented with L-glutamine, 10% FBS, and penicillin (100 U/ml)-streptomycin (100 µg/ml). Cells were maintained at 37 °C in a humidified incubator with 5% CO₂ and routinely cultured at a density ranging from 2 × 10^5^ to 1.5 × 10⁶ cells/ml. The number of passages did not exceed ten. Unless otherwise indicated, cells were seeded at a density of 7.5 × 10^4^ cells/ml and treated for 48 h with various concentrations of potassium clavulanate freshly prepared in complete RPMI 1640 medium.

The 293T cell line used for lentiviral packaging was purchased from Biosettia (San Diego, CA) and maintained in DMEM supplemented with 10% FBS, 2 mM L-glutamine, 1 mM sodium pyruvate, 100 µM non-essential amino acids, and penicillin (100 U/ml)-streptomycin (100 µg/ml). Cells were cultured at 37 °C in a humidified atmosphere containing 5% CO₂.

### Generation of TK6 cells overexpressing human CYP isoforms

The coding sequences of 18 individual human CYP isoforms were cloned into the lentiviral expression vector pLV-EF1a-IRES-Bsd, as described in our previous study^18^. The procedures for generating lentiviral stocks containing either human CYP expression vectors or empty vectors, as well as for generating CYP-expressing TK6 cell lines, were also detailed in that study^18^. Briefly, TK6 cells were seeded into 6-well tissue culture plates at a volume of 2 ml/well at a density of 2.5 × 10^5^ cells/ml and infected with lentivirus at a multiplicity of infection of 10 in the presence of 8 µg/ml polybrene (Biosettia). Viral transduction was enhanced by centrifugation at 1000 × g for 1 h at room temperature. After centrifugation, the transduction medium was replaced with fresh complete medium. At 48 h post-transduction, cells were selected with 6 μg/ml blasticidin to generate blasticidin-resistant TK6 cell lines stably expressing individual human CYP isoforms (TK6/CYP) or the empty vector control (TK6/vector).

### Western blot analysis

TK6/CYP, TK6/vector, and TK6 cells (1 × 10⁷ cells) were lysed in RIPA buffer for 30 min on ice, followed by sonication three times for 10 s. The lysates were centrifuged at 14,000 × g for 30 min at 4 °C, and the resulting supernatants were collected as whole-cell lysates. Protein concentrations were determined using a BCA protein assay (Thermo Fisher Scientific, Inc.). Equivalent amounts of protein (20 μg) were separated on 10% Bis-Tris SDS-polyacrylamide gels and transferred onto PVDF membranes. Membranes were blocked with 5% nonfat milk and incubated with primary antibodies against CYP1A1, CYP1A2, CYP1B1, CYP2A6, CYP2A7, CYP2A13, CYP2B6, CYP2C8, CYP2C9, CYP2C18, CYP2C19, CYP2D6, CYP2E1, CYP3A4, CYP3A5, CYP3A7, CYP4A11, CYP4B1, γ-H2AX, and β-actin (dilution 1:500 - 1:2000 in 5% nonfat milk), followed by incubation with HRP-conjugated secondary antibodies. These primary antibodies have been previously characterized^18^. Protein bands were detected by chemiluminescence using Immobilon Western HRP substrate (Millipore, Billerica, MA) and imaged with the FluoroChem E system and AlphaView software (ProteinSimple, San Jose, CA). Protein bands were quantified using AlphaView software under non-saturating conditions. Original blots are provided in Supplementary Information File 1.

### CYPs activity assay

For each cell line, 2 × 10^8^ cells were harvested, suspended in 6 ml of ice-cold microsome preparation buffer (250 mM sucrose, 10 mM Tris, pH 7.4), and subjected to microsome preparation as previously described^18^. Enzymatic activities of CYPs in microsomes isolated from parental TK6 cells, TK6/vector control cells, and the 18 TK6/CYP-overexpressing lines were evaluated using the known substrate triclosan^18^, except for CYP2A6, CYP4A11, CYP2C8, and CYP2C9. The activities of these CYPs in the corresponding overexpressing cells were assessed using the P450-Glo™ assay (Promega, Madison, WI), as previously described^18^.

### Half-maximal inhibitory concentration (IC_50_) of clavulanic acid on cell growth

The IC_50_ values of clavulanic acid in TK6/CYP, TK6/vector, and parental TK6 cells were determined using the CellTiter-Glo Luminescent Cell Viability Assay (Promega) according to the manufacturer’s instructions. Briefly, cells were seeded into 96-well plates at a volume of 100 μl/well at densities of 2.0 × 10^5^ and 7.5 × 10^4^ cells/ml and incubated with freshly prepared potassium clavulanate (20 - 1000 μM) in RPMI1640 medium for 24 and 48 h, respectively. Control cells were cultured in complete medium only. At the end of the treatment period, CellTiter-Glo® Reagent was added to the cells at a 1:1 ratio, and the mixtures were transferred to the wells of a white flat-bottom 96-well plate in quadruplicate. Luminescent signals were measured immediately using a BioTek Cytation 5 cell imaging multimode reader (BioTek Instruments, Inc., Winooski, Vermont). The luminescent signals observed in clavulanic acid-treated cells were normalized relative to those in control cells. IC_50_ values were calculated from the cell growth curves using GraphPad Prism 6.0 (GraphPad Software, La Jolla, CA).

### Cytotoxicity of clavulanic acid in TK6/vector and TK6/CYP3A4 cells

A CellTiter-Glo Luminescent Cell Viability Assay kit (Promega) was used to measure the cell viability. Briefly, cells were cultured in 24-well plate at a volume of 500 µl/well at 7.5 × 10^4^ cells/ml, and then treated with 0.1 x, 0.5 x, and 1.0 x IC_50_ of freshly prepared potassium clavulanate for 48 h. Control cells were cultured in complete medium only. After 48 h, aliquots (50 µl) of cells were dispensed into the wells of a white flat-bottom 96-well plate in quadruplicate and mixed with CellTiter-Glo® Reagent at a ratio of 1:1. Luminescence was measured immediately with a BioTek Cytation 5 cell imaging multimode reader. The relative cell viability was calculated by comparing the luminescence intensity of the treated cells to that of the controls. An LDH assay was used to assess necrotic cell death as describe previously^19^. LDH release was defined as the LDH activity measured in the culture media following drug treatment compared to that measured the total cell lysate for each sample.

### The apoptosis assay

Apoptotic cell death was quantified using the FITC Annexin V Apoptosis Detection Kit I (BD Biosciences, San Jose, CA) following 48 h of potassium clavulanate treatment. Briefly, cells were collected and washed twice with cold PBS and then resuspended in binding buffer at a concentration of 1 × 10^6^ cells/ml. One hundred microliters of cell suspension were incubated with 5 μl FITC Annexin V and 5 μl propidium iodide for 15 min at room temperature in the dark. The stained cells were diluted with 400 μl of binding buffer and analyzed on a FACSCanto II flow cytometer (BD Biosciences).

### EdU proliferation assay

A 5-ethynyl-2′-deoxyuridine (EdU) assay was used to detect and quantify cell proliferation by measuring newly synthesized DNA. The assay was performed using a Click-iT® EdU Flow Cytometry Assay kit (Thermo Fisher Scientific) as previously described^20^. Briefly, after 48 h of exposure to potassium clavulanate, cells were incubated with 10 μM EdU for 2 h. Cells were then harvested, adjusted to 1 × 10⁶ cells/sample, and subjected to EdU staining and flow cytometric analysis according to a published protocol^20^.

### Cell cycle analysis

Cell cycle progression was assessed by flow cytometry as previously described^21^. Cells were exposed to potassium clavulanate for 48 h, followed by incubation with 10 μM BrdU for 1 h. Cells were then harvested, fixed in 70% ethanol, and stained with anti-BrdU-FITC monoclonal antibody and propidium iodide. The stained cells were analyzed using a Beckman Coulter CytoFlex flow cytometer (Indianapolis, IN) with CytExpert 1.2 software (Beckman Coulter).

### MultiFlow DNA damage assay

A MultiFlow DNA Damage kit (Litron Laboratories, Rochester, NY) was used to assess DNA damage response pathways. Cells were exposed to potassium clavulanate for 48 h, resuspended in PBS, and 25 μl of the suspension was added to wells of a 96-well round-bottom plate containing 50 μl of complete labeling solution per well, prepared according to the manufacturer’s instructions. The labeling solution contained anti-γH2AX-Alexa Fluor 647, anti-phospho-histone H3-PE, and anti-p53-FITC antibodies to measure DNA double-strand breaks, mitotic cells, and p53 activation, respectively. After a 1-h incubation at room temperature, the cells were analyzed using a BD FACSCanto II Cell Analyzer equipped with a high throughput sampler (BD Biosciences) according to a published protocol^22^.

### Statistical analyses

Data are presented as the mean ± standard deviation of data from at least three independent experiments. The data were analyzed by a two-way ANOVA, with pairwise comparisons to the control samples made by Dunnett’s multiple comparisons test (GraphPad 6.0). Differences were considered statistically significant at p < 0.05.

## Results

### Generation of TK6-derived cell lines with CYP overexpression

**Fig. 2:**
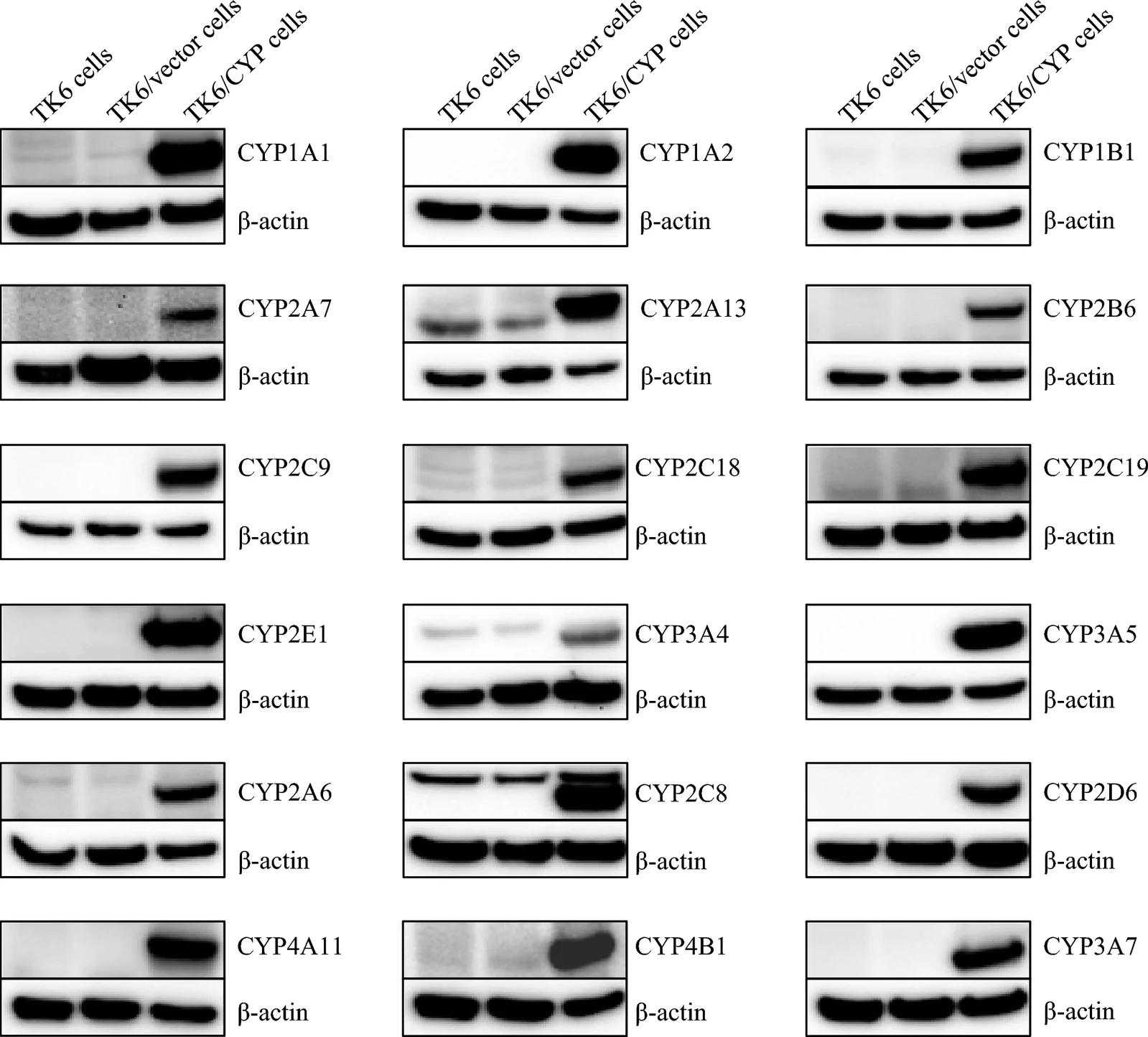
Western blot analysis of individual CYP isoform expression in TK6/CYP-overexpressing cells. β-Actin was used as the loading control.

To investigate the impact of CYP-mediated metabolism on the cytotoxicity of clavulanic acid, a panel of TK6-derived cell lines was generated, each overexpressing a single human CYP isoform, including CYP1A1, CYP1A2, CYP1B1, CYP2A6, CYP2A7, CYP2A13, CYP2B6, CYP2C8, CYP2C9, CYP2C18, CYP2C19, CYP2D6, CYP2E1, CYP3A4, CYP3A5, CYP3A7, CYP4A11, and CYP4B1. Western blot analysis demonstrated that none of the CYP isoforms examined was expressed in parental TK6 or TK6/vector control cells (Fig. 2). In contrast, robust expression of each CYP isoform was detected in the corresponding TK6/CYP-overexpressing cell lines (Fig. 2).

To confirm further the metabolic functionality of CYPs in the newly generated TK6/CYP-overexpressing cells, microsomes were isolated from parental TK6 cells, TK6/vector control cells, and the 18 TK6/CYP-overexpressing lines. As shown in Table 1**,** all CYP isoforms exhibited enzymatic activities toward their respective known substrates, except CYP2A7, whose activity could not be evaluated due to the lack of commercially available assay kits or identified substrates.

**Table 1:** CYP enzymatic activities^a^.

Cell lines	2,4-Dichlorophenol formation (pmol min^−1^ mg protein^−1^)	P450-Glo™ assay (fold of TK6/vector cells)^b^
TK6	n.d.	
Vector	n.d.	1.0
CYP1A1	0.39 ± 0.06	
CYP1A2	9.00 ± 0.26	
CYP1B1	0.72 ± 0.05	
CYP2A6	n.d.	170.2 ± 4.1
CYP2A7	n.d.	n/a
CYP2A13	0.45 ± 0.03	
CYP2B6	3.13 ± 1.08	
CYP2C8	n.d.	34.3 ± 1.1
CYP2C9	n.d.	35.7 ± 2.1
CYP2C18	0.77 ± 0.10	
CYP2C19	1.59 ± 1.08	
CYP2D6	1.02 ± 0.11	
CYP2E1	0.14 ± 0.05	
CYP3A4	0.72 ± 0.07	
CYP3A5	0.14 ± 0.07	
CYP3A7	0.36 ± 0.02	
CYP4A11	n.d.	146.5 ± 10.2
CYP4B1	0.10 ± 0.03	

^a^CYP enzymatic activities in microsomes isolated from parental TK6 cells, TK6/vector cells, and TK6/CYP-overexpressing cells were evaluated using the known substrate triclosan^18^, except for CYP2A6, CYP2C8, CYP2C9, and CYP4A11. The activities of these CYP isoforms in the corresponding overexpressing cells were measured using the P450-Glo™ assay (Promega), as previously described^18^.

^b^CYP enzymatic activity was not detected in microsomes from TK6/vector cells, as the luminescence values were similar to those of the reagent controls. Relative CYP enzymatic activities of CYP2A6, CYP2C8, CYP2C9, and CYP4A11 were calculated using the luminescence values from TK6/vector cell microsomes as the reference.

Data are presented as the mean ± standard deviation. n.d., not detectable; n/a, not determined due to the lack of commercially available assay kits.

### IC_50_ values of clavulanic acid on cell growth

**Fig. 3:**
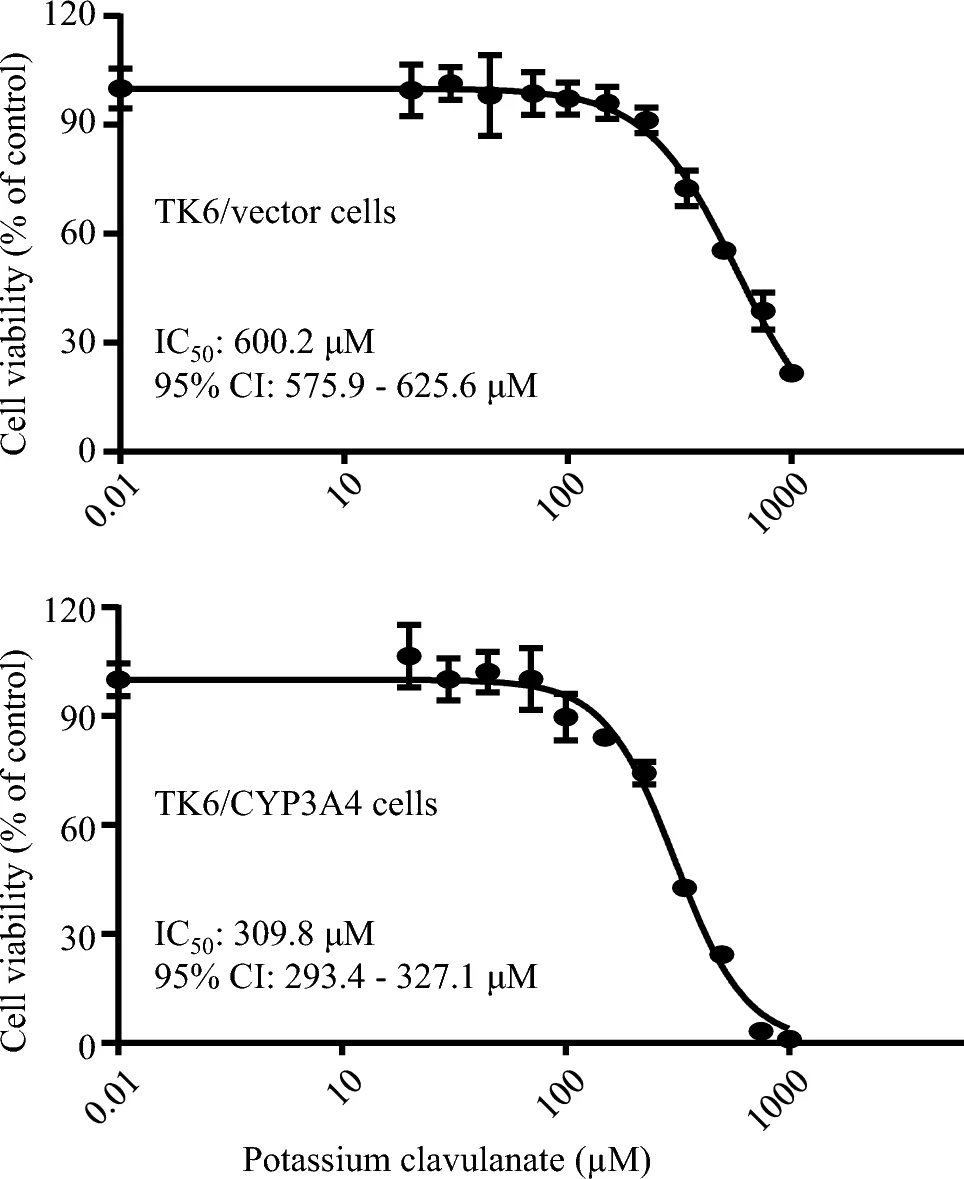
Representative cell growth curves of TK6/vector and TK6/CYP3A4 cells. Cells were incubated with freshly prepared potassium clavulanate (20 - 1000 μM) in RPMI 1640 medium for 48 h. After exposure to clavulanic acid, cell viability was measured using an ATP assay as described in the Materials and Methods. Cell growth curves were generated using GraphPad Prism 6.0. CI: Confidence intervals.

The effects of clavulanic acid on cell growth were assessed; representative growth curves are presented in Fig. 3. Clavulanic acid inhibited cell growth in a concentration-dependent manner, with IC₅₀ values being substantially higher following 24 h of exposure compared with 48 h (Table 2). Similar IC₅₀ values were observed in parental TK6 cells, TK6/vector control cells, and five CYP-overexpressing cell lines (CYP2A6, CYP2A7, CYP2C8, CYP3A5, and CYP4B1). In contrast, 13 CYP-overexpressing cell lines showed lower IC₅₀ values than controls. The rank order was CYP3A4 ≈ CYP3A7 ≈ CYP1A1 < CYP4A11 ≈ CYP2A13 ≈ CYP2C19 < CYP2B6 ≈ CYP1A2 ≈ CYP2C9 < CYP1B1 ≈ CYP2D6 < CYP2E1 ≈ CYP2C18, with the greatest reduction (~2-fold) observed in TK6/CYP3A4 cells (Table 2).

**Table 2:** Half-maximal inhibitory concentration (IC_50_) of potassium clavulanate on cell growth^a^

Cell lines	IC_50_ (μM)		95% confidence intervals (μM)	
	24 h	48 h	24 h	48 h
TK6	1314	555.8	1088–1586	528.0 – 584.9
TK6/vector	1296	600.2	1126–1492	575.9 – 625.6
TK6/CYP1A1	768.7*	337.2*	720.2 – 820.4	318.0 – 357.5
TK6/CYP1A2	1103	388.6*	992.0 – 1227	373.8 – 406.3
TK6/CYP1B1	903.7*	443.4*	850.9 – 959.9	412.4 – 476.8
TK6/CYP2A6	1267	606.4	997.1 – 1611	578.6 – 635.6
TK6/CYP2A7	1232	631.8	1084 – 1401	591.4 – 675.0
TK6/CYP2A13	984.6*	364.9*	893.6 – 1085	337.6 – 394.3
TK6/CYP2B6	961.6*	378.8*	879.3 – 1052	355.3 – 403.8
TK6/CYP2C8	1121	628.6	1012 – 1241	604.9 – 653.2
TK6/CYP2C9	803.7*	392.0*	729.7 – 885.3	370.8 – 414.4
TK6/CYP2C18	1099	490.7*	994.7 – 1214	472.0 – 510.1
TK6/CYP2C19	964.8*	367.7*	902.1 – 1032	345.9 – 390.7
TK6/CYP2D6	995.1*	444.6*	925.1 – 1070	425.5 – 464.5
TK6/CYP2E1	971.1*	476.9*	875.2 – 1078	454.1 – 500.9
TK6/CYP3A4	842.5*	309.8*	777.5 – 913.0	293.4 – 327.1
TK6/CYP3A5	1386	557.7	1010 – 1901	526.4 – 591.0
TK6/CYP3A7	707.7*	332.2*	671.9 – 745.4	317.8 – 347.3
TK6/CYP4A11	1017	353.6*	943.6 – 1095	331.7 – 376.9
TK6/CYP4B1	1131	532.9	1027 – 1246	508.0 – 559.1

^a^Cells were incubated with various concentrations (20 – 1000 μM) of potassium clavulanate freshly dissolved in RPMI1640 medium for 24 or 48 h. The IC_50_ values were calculated from the cell growth curves using GraphPad Prism 6.0. Data are presented as the mean IC_50_ values from three independent experiments with 95% confidence intervals. *: a significant difference (p < 0.05) was observed compared with TK6 parental cells and TK6/vector cells.

### Effects of clavulanic acid on cell viability

**Fig. 4:**
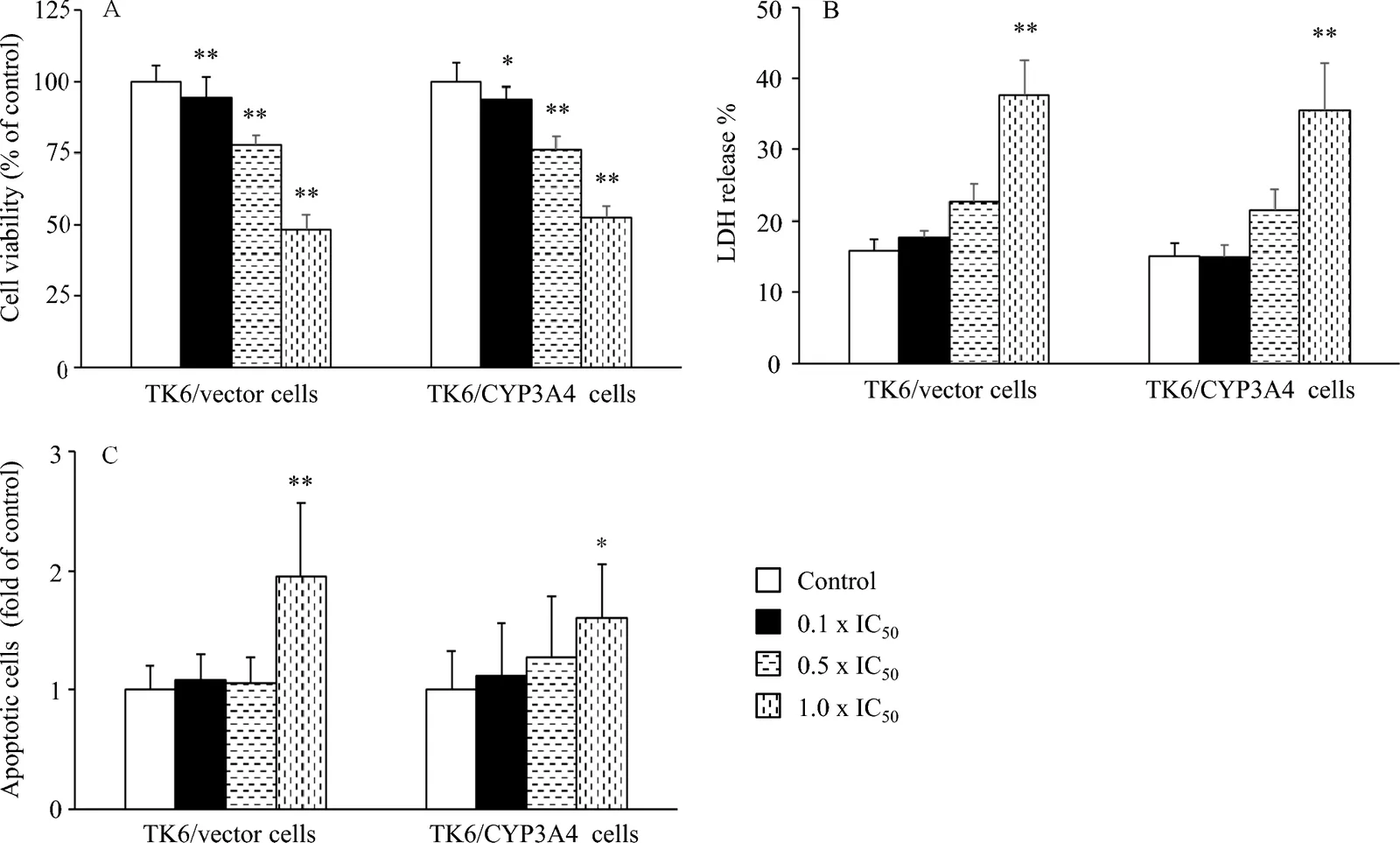
Cell viability, necrosis, and apoptosis in TK6/vector and TK6/CYP3A4 cells following exposure to clavulanic acid. (**A**) Cell viability was assessed by an ATP assay, (**B**) necrotic cell death was evaluated by an LDH release assay, and (**C**) Apoptotic cells were quantified using an annexin V-based apoptosis detection assay in combination with propidium iodide staining. Clavulanic acid was added at culture initiation, and cells were exposed to 0, 0.1×, 0.5×, or 1.0× IC₅₀ concentrations for 48 h. Data are presented as the mean ± SD of three independent experiments. *: Significantly different from untreated controls (*p < 0.05; **p < 0.01).

To characterize further the contribution of CYP-mediated metabolism to the cytotoxicity of clavulanic acid, its effects were evaluated in TK6/vector and TK6/CYP3A4 cells. Based on IC₅₀ values for cell growth, equitoxic concentrations corresponding to 0.1×, 0.5×, and 1.0× IC₅₀ were selected. These concentrations were 60, 300, and 600 µM potassium clavulanate for TK6/vector cells and 31, 105, and 310 µM potassium clavulanate for TK6/CYP3A4 cells. The concentrations of clavulanate used in this study exceeded reported peak plasma levels in humans (approximately 5 - 30 µM)^23^ and were selected to enable mechanistic evaluation of CYP-dependent cytotoxicity and DNA damage responses under controlled *in vitro* conditions. Cell viability at these concentrations was first assessed in both cell lines. After 48 h of exposure, a similar and significant decrease in cell viability was observed in both cell types (Fig. 4A**)**. Compared with control cultures, growth rates at 0.1×, 0.5×, and 1.0× IC₅₀ were 94.5%, 77.8%, and 48.2% in TK6/vector cells and 93.9%, 76.2%, and 52.3% in TK6/CYP3A4 cells, respectively. The observed reduction in cell viability may be attributable to increased necrotic cell death, induction of apoptosis, and/or alterations in cell cycle progression. Each of these mechanisms was subsequently investigated.

### Effect of clavulanic acid on necrotic cell death

To determine whether the observed decrease in viable cell numbers was attributable to necrotic cell death, LDH release was measured. LDH release assays are widely used as a marker of cell death^24^, as the release of LDH into the culture medium accurately reflects necrotic cell death *in vitro*. After 48 h of exposure, a similar and concentration-related trend increase in LDH release was observed in both cell lines (Fig. 4B). In TK6/vector cells, LDH release was 15.7%, 17.7%, 22.7%, and 37.6%, whereas in TK6/CYP3A4 cells, the corresponding values were 15.1%, 14.9%, 21.5%, and 35.6% following treatment with 0, 0.1×, 0.5×, and 1.0× IC₅₀ clavulanic acid, respectively. A statistically significant increase was observed only at 1.0× IC₅₀ clavulanic acid. The elevated LDH release indicates that the concentrations of clavulanic acid used were cytotoxic and suggests that the reduction in viable cells may be partially attributable to necrotic cell death.

### Effect of clavulanic acid on apoptotic cell death

To determine whether clavulanic acid induced apoptosis after 48 h of treatment, Annexin V/PI staining followed by flow cytometric analysis was performed. Both cell lines exhibited a similar concentration-related trend toward increased apoptosis (Fig. 4C). In TK6/vector cells, the percentage of apoptotic cells was 11.2%, 12.2%, 11.8%, and 21.8%, whereas in TK6/CYP3A4 cells, the corresponding values were 11.5%, 12.9%, 14.6%, and 18.3% following treatment with 0, 0.1×, 0.5×, and 1.0× IC₅₀ clavulanic acid, respectively. A statistically significant increase was observed only at 1.0× IC₅₀ clavulanic acid. These findings suggest that apoptosis contributes to the reduction in viable cell numbers at higher concentrations of clavulanic acid.

### Effect of clavulanic acid on cell cycle progression and DNA synthesis

**Fig. 5:**
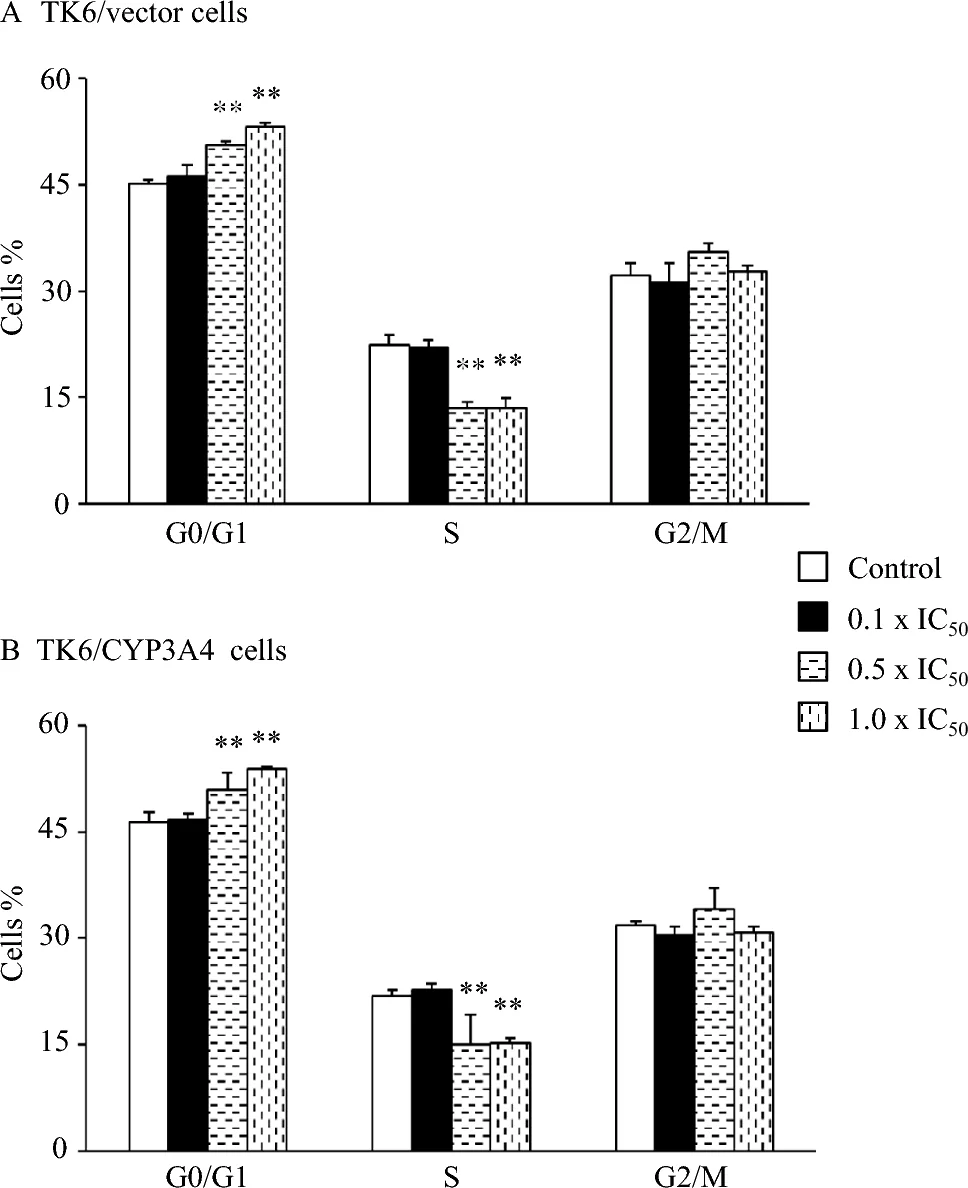
Cell cycle analysis in TK6/vector and TK6/CYP3A4 cells following exposure to clavulanic acid. (**A**) TK6/CYP vector cells and (**B**) TK6/CYP3A4 cells were examined using an anti-BrdU incorporation assay to quantify the cell cycle distribution. Clavulanic acid was added at culture initiation, and cells were treated with 0, 0.1×, 0.5×, or 1.0× IC₅₀ concentrations for 48 h. Data represents the mean ± SD from three independent experiments. *: Significantly different from untreated controls (**p < 0.01).

The effect of clavulanic acid on cell cycle progression was evaluated by flow cytometry. In TK6/vector cells, treatment with clavulanic acid for 48 h at the two highest concentrations resulted in a significant increase in the proportion of cells in the G0/G1 phase, accompanied by a corresponding decrease in the proportion of cells in S phase (Fig. 5A). Specifically, the percentage of cells in G0/G1 phase increased from 45.3% in control cultures to 50.5% and 53.2% at 300 and 600 µM clavulanic acid, respectively, while the percentage of cells in S phase decreased from 22.4% to 13.4% and 13.5%. No significant changes were observed in the G2/M phase.

Similarly, TK6/CYP3A4 cells exhibited a significant increase in the proportion of cells in the G0/G1 phase at the two highest concentrations of clavulanic acid, rising from 46.4% in control cultures to 50.8% and 53.9%, respectively (Fig. 5B). Concomitant with the increase in G0/G1 phase cells, treatment at these concentrations of clavulanic acid resulted in a significant reduction in the proportion of cells in S phase, decreasing from 21.8% to 15.0% and 15.2%, respectively, with no significant changes in the G2/M phase (Fig. 5B). These findings indicate that clavulanic acid–mediated cell growth inhibition may be associated with G0/G1 phase cell cycle arrest.

**Fig. 6:**
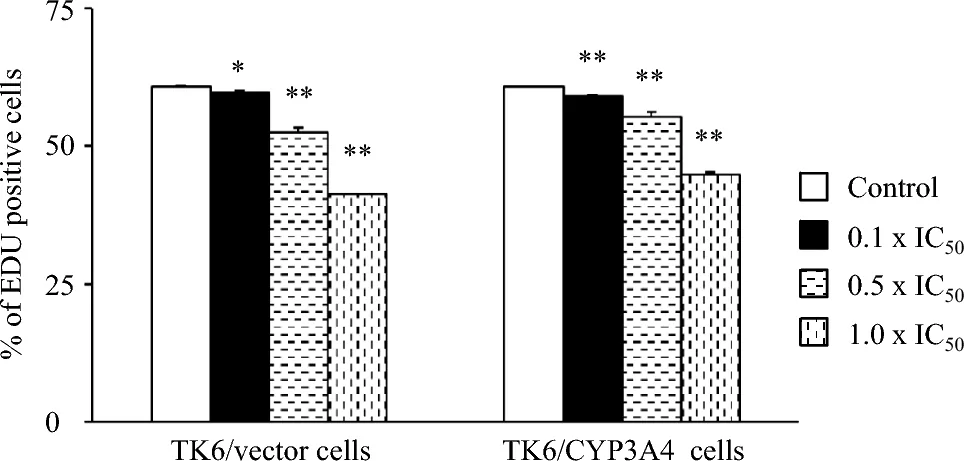
Proliferation analysis in TK6/vector and TK6/CYP3A4 cells following exposure to clavulanic acid, as determined by an EdU incorporation assay. Clavulanic acid was added at the initiation of cultures, and cells were treated with 0, 0.1×, 0.5×, or 1.0× IC₅₀ concentrations for 48 h. Data represent the mean ± SD from three independent experiments. *: Significantly different from untreated controls (*p < 0.05; **p < 0.01).

To elucidate further the mechanism underlying cell cycle disruption, DNA synthesis during S phase was assessed using an EdU incorporation assay. As shown in Fig. 6, after 48 h of exposure, both cell lines exhibited a concentration-dependent decrease in the proportion of EdU-positive cells, with a significant difference being observed between TK6/vector and TK6/CYP3A4 cells. These findings indicate reduced DNA synthesis, consistent with the decreased proportion of cells in S phase.

### Effects of clavulanic acid on γH2AX expression

**Fig. 7:**
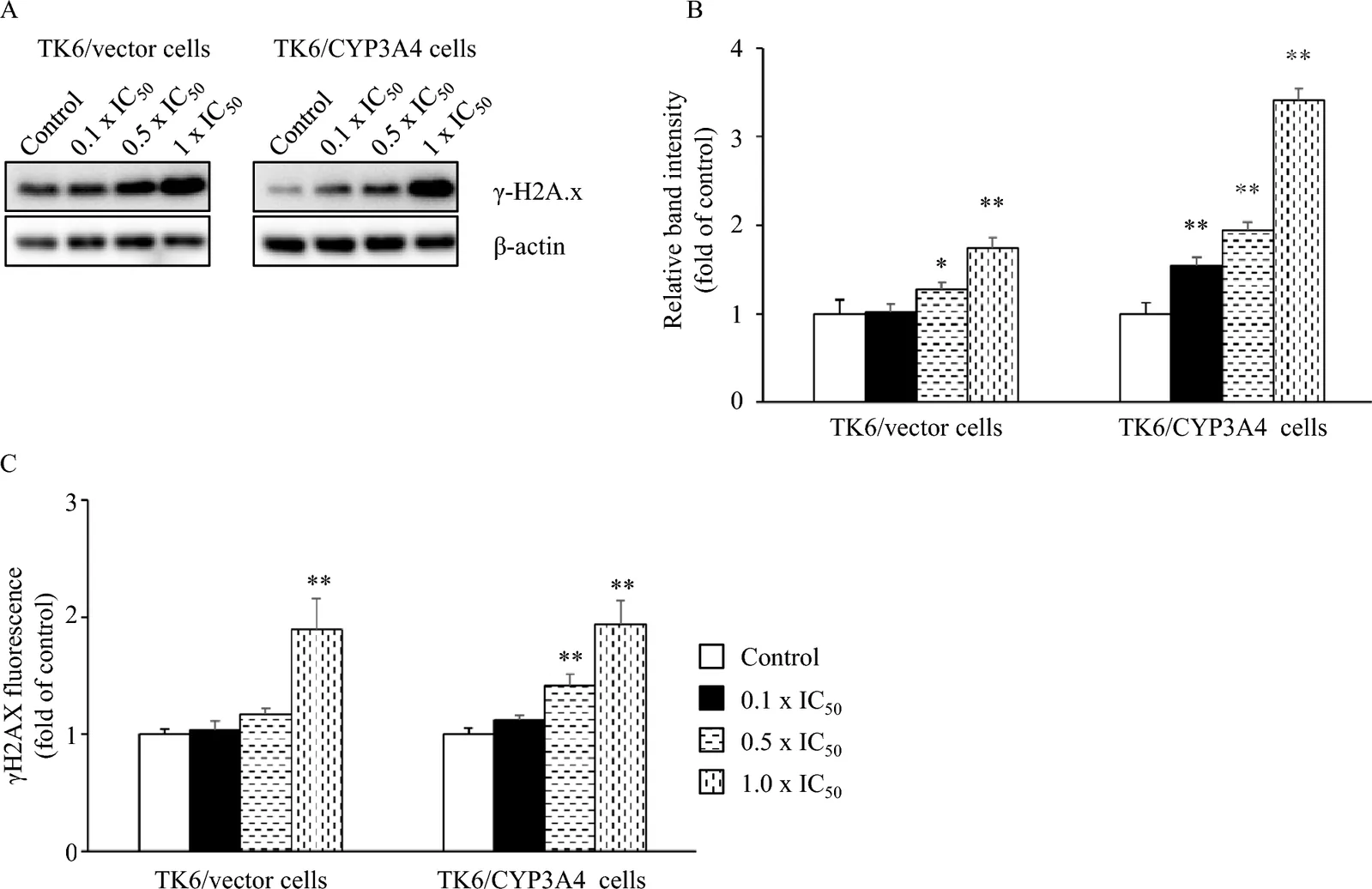
Induction of γH2AX in TK6/vector and TK6/CYP3A4 cells following exposure to clavulanic acid. Total cellular proteins were isolated 48 h after treatment with 0, 0.1x, 0.5x, and 1.0x IC_50_ concentrations of clavulanic acid. γH2AX levels were determined by Western blot analysis, with β-actin serving as the internal loading control. Band intensities were quantified, and relative protein levels were normalized to β-actin. (**A**) Representative immunoblots. (**B**) Relative level of γH2A.x from three independent experiments. (**C**) γH2A.x expression was further evaluated using a MultiFlow assay, with results representing three independent experiments. *: Significantly different from untreated controls (*p < 0.05; **p < 0.01).

The observed cell cycle disturbances and apoptosis may be related to DNA damage. To assess whether clavulanic acid induces DNA damage, γH2AX expression, a marker of DNA damage, was evaluated by Western blot. As shown in Fig. 7A and B, treatment with clavulanic acid for 48 h resulted in a significant difference in γH2AX induction between TK6/vector and TK6/CYP3A4 cells. In TK6/vector cells, γH2AX levels increased in a concentration-related trend manner, with significant elevations at 300 and 600 µM. By comparison, a greater concentration-dependent increase in γH2AX (p < 0.01) was observed in TK6/CYP3A4 cells across all treatment concentrations, indicating greater DNA damage. The increase in γH2AX was further confirmed by elevated γH2AX fluorescence in both cell lines using a MultiFlow flow cytometry–based assay after 48 h of treatment with equitoxic concentrations of clavulanic acid (Fig. 7C). The MultiFlow assay did not detect changes in other biomarkers, including phosphorylated histone H3 or nuclear p53 (data not shown). Together, these results suggest that clavulanic acid induces γH2AX, likely reflecting DNA damage associated with its cytotoxicity.

## Discussion

In the present study, we investigated the role of CYP-mediated metabolism in clavulanic acid-induced cytotoxicity using a panel of TK6-derived human lymphoblastoid cell lines individually overexpressing human CYP isoforms. This experimental system enabled systematic evaluation of isoform-specific metabolic contributions in a genetically consistent background lacking endogenous CYP activity, thereby allowing direct attribution of altered toxicological responses to CYP-dependent metabolism.

All engineered TK6/CYP cell lines exhibited robust expression and enzymatic activity of their respective CYP isoforms, whereas parental TK6 and vector control cells showed no detectable CYP expression or activity, consistent with previous reports^25,26^. This validation supports the conclusion that differences in clavulanic acid sensitivity observed among the cell lines reflect CYP-dependent modulation rather than nonspecific cellular alterations introduced during cell line generation.

Clavulanic acid inhibited cell growth in a concentration- and time-dependent manner across all cell lines examined. Importantly, overexpression of the majority of CYP isoforms tested resulted in reduced IC₅₀ values compared with parental and vector control cells, indicating that CYP-mediated metabolism generally enhances the cytotoxic potency of clavulanic acid. Among these isoforms, CYP3A4 produced the most pronounced effect, reducing the IC₅₀ approximately two-fold. Given the broad substrate specificity and high hepatic abundance of CYP3A4^15^, this result suggests that CYP3A4-mediated metabolism enhances the intrinsic cytotoxic potential of clavulanic acid.

Despite its limited intrinsic toxicity, clavulanic acid has been strongly implicated in DILI, especially when administered with amoxicillin^7,13^. Importantly, clinical and experimental evidence suggests that clavulanic acid is the principal driver of this liver injury^6,8^. The present findings provide mechanistic support for a metabolism-dependent contribution to clavulanate-associated toxicity. Specifically, the enhanced cytotoxicity observed in CYP-overexpressing cell lines reflects CYP-dependent modulation of toxic potency rather than definitive evidence of metabolic bioactivation, as specific metabolites or reactive intermediates were not directly measured. Nevertheless, the consistent reduction in IC₅₀ values across multiple CYP isoforms is compatible with increased formation of cytotoxic species or altered intracellular handling of clavulanic acid, mechanisms commonly implicated in metabolism-dependent adverse drug reactions^27^.

Despite the increased growth inhibition observed in CYP3A4-overexpressing cells, cytotoxic endpoints were largely comparable between TK6/vector and TK6/CYP3A4 cells when assessed at equitoxic concentrations. Both cell lines exhibited similar reductions in cell viability, increases in LDH release, the induction of apoptosis at higher concentrations, and disruption of cell cycle progression. LDH release, a widely accepted indicator of necrotic cell death^24^, increased to a similar extent in both cell lines, indicating that loss of membrane integrity contributes to clavulanic acid–induced cytotoxicity independent of CYP3A4 expression once cytotoxic thresholds are exceeded. These findings suggest that CYP3A4 primarily influences the intracellular concentration required to initiate cytotoxicity rather than altering the qualitative nature of the ensuing cell death response.

Apoptotic cell death was detected predominantly at the highest concentrations of clavulanic acid, indicating that programmed cell death pathways are engaged under conditions of substantial cellular stress. In addition to cell death, clavulanic acid significantly perturbed cell cycle progression, inducing G0/G1 phase arrest accompanied by a reduction in S-phase cells in both CYP-deficient and CYP3A4-expressing cells. This effect was further supported by reduced EdU incorporation, demonstrating impaired DNA synthesis. Such cell cycle arrest is a characteristic response to genotoxic or replication interference and represents a common adaptive response aimed at preventing the propagation of damaged DNA^28^.

Consistent with these observations, clavulanic acid exposure increased the levels of γH2AX, a sensitive marker of DNA damage signaling associated with double-strand breaks and replication stress^29,30^. Notably, γH2AX induction occurred in a concentration-dependent manner in TK6/CYP3A4 cells, whereas significant induction in TK6/vector cells was observed only at the highest concentration tested. This pattern suggests that CYP3A4-mediated metabolism enhances DNA damage signaling, potentially by increasing intracellular exposure to cytotoxic species. However, it should be noted that γH2AX phosphorylation may also arise secondary to replication stress or apoptosis-associated DNA fragmentation. Accordingly, the present findings indicate activation of DNA damage response pathways but do not unequivocally establish the primary toxicity.

The absence of detectable changes in phosphorylated histone H3 or nuclear p53 further suggests that, under the experimental conditions employed, clavulanic acid–induced stress preferentially activates early DNA damage signaling without fully engaging mitotic disruption or canonical p53-dependent responses. Together, these results support a model in which CYP-mediated metabolism, particularly via CYP3A4, lowers the threshold for clavulanic acid–induced cytotoxicity by enhancing DNA damage signaling and cell cycle arrest, while the downstream mechanisms of necrosis and apoptosis remain largely conserved once cytotoxicity is established.

Several limitations of this study should be acknowledged. CYP expressions and activity in TK6/CYP-overexpressing cells may not fully reflect those in freshly isolated human hepatocytes, as this model was designed primarily to evaluate the contribution of individual CYP isoforms to chemical-induced cytotoxicity. Variations in cellular background, CYP regulation, and metabolic capacity may influence enzyme activity; thus, the findings should be considered as qualitative and comparative rather than representative of *in vivo* hepatic metabolism. Moreover, direct identification of clavulanic acid metabolites and reactive intermediates was not performed, limiting definitive conclusions regarding specific bioactivation pathways, and oxidative stress was not evaluated. Although TK6 cells provide a genetically stable system lacking endogenous CYP activity, they do not capture the full metabolic complexity of hepatocytes. Consequently, extrapolation of these findings to *in vivo* liver injury should be made with caution, and further studies using metabolically competent hepatic models are warranted to better define the toxicological relevance of CYP-mediated clavulanic acid metabolism.

In conclusion, this study demonstrates that CYP enzymes modulate clavulanic acid-induced cytotoxicity in an isoform-dependent manner, with CYP3A4 exerting the strongest effect on cytotoxic potency. These findings provide mechanistic insight into metabolism-dependent modulation of clavulanate toxicity and may help explain interindividual variability in susceptibility to clavulanate-associated adverse effects. Further characterization of CYP-generated metabolites and their molecular targets will be essential for advancing understanding of clavulanic acid-associated drug-induced toxicity.

## Supplementary Information

Supplementary Information.

## Data Availability

The datasets used and/or analyzed during the current study are available from the corresponding author upon reasonable request.
