# Supplementary material for: Contribution of cytochrome P450-mediated metabolism to clavulanic acid-induced cytotoxicity in TK6-derived cytochrome P450-overexpressing cell lines
Source: Sci Rep. 2026 Jul 15;16:32365. doi: 10.1038/s41598-026-59728-9 (PMC13668705; doi:10.1038/s41598-026-59728-9)
Supplement: Supplementary file 1 [file 41598_2026_59728_MOESM1_ESM.pdf]

Figure 2

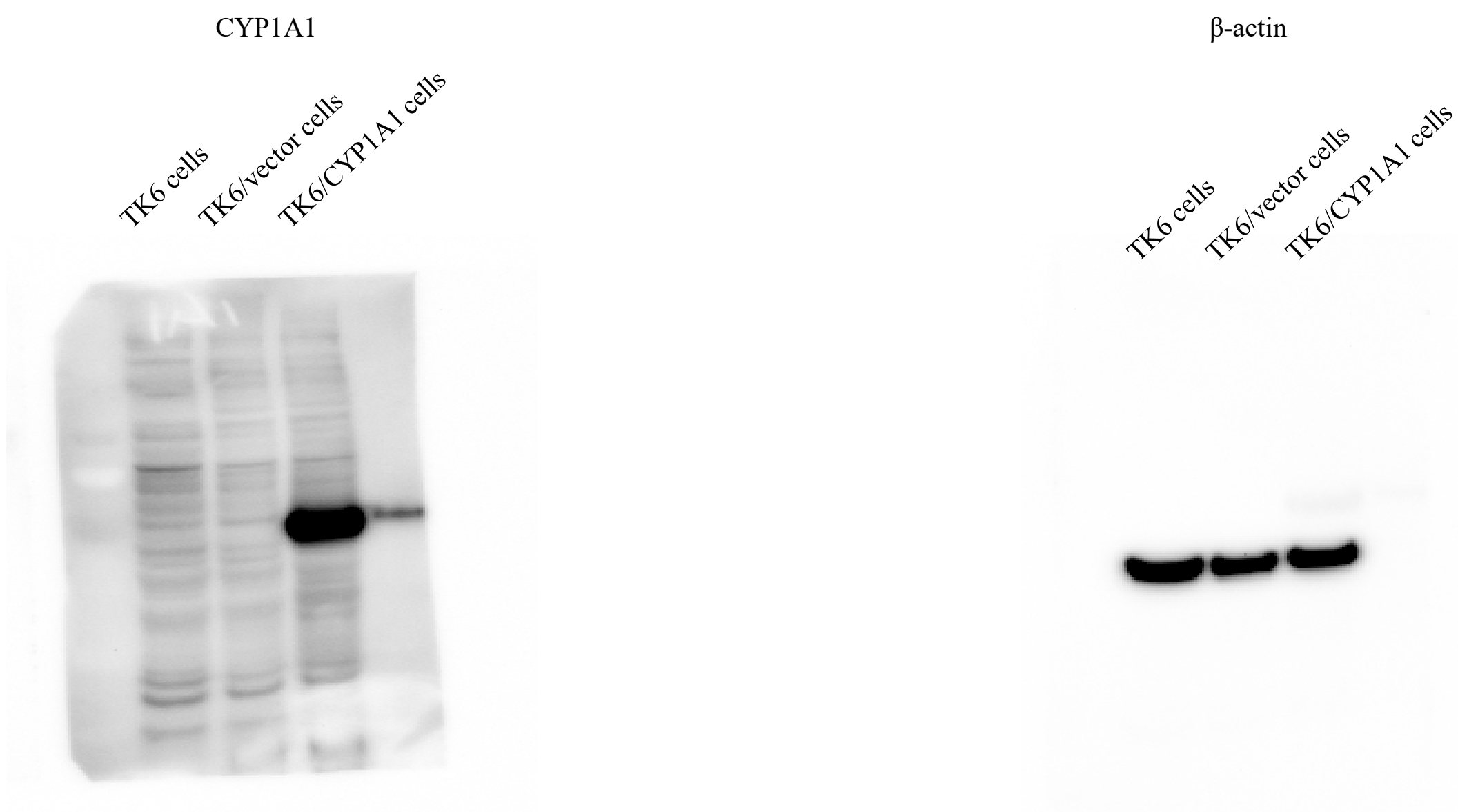
CYP1A1
TK6 cells
TK6/vector cells
TK6/CYP1A1 cells
β-actin
TK6 cells
TK6/vector cells
TK6/CYP1A1 cells

Figure 2

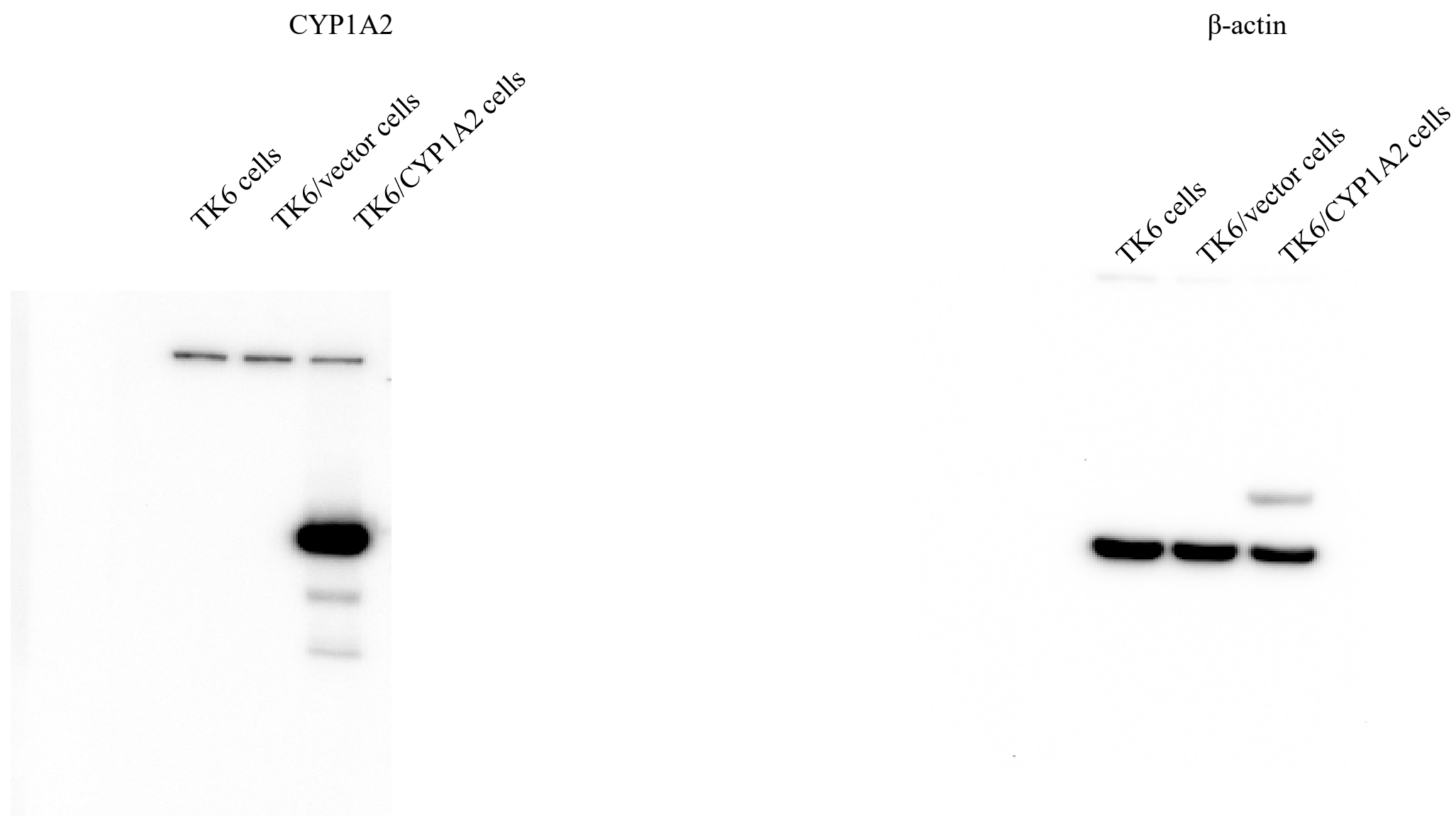

CYP1A2
TK6 cells
TK6/vector cells
TK6/CYP1A2 cells
β-actin
TK6 cells
TK6/vector cells
TK6/CYP1A2 cells

Figure 2

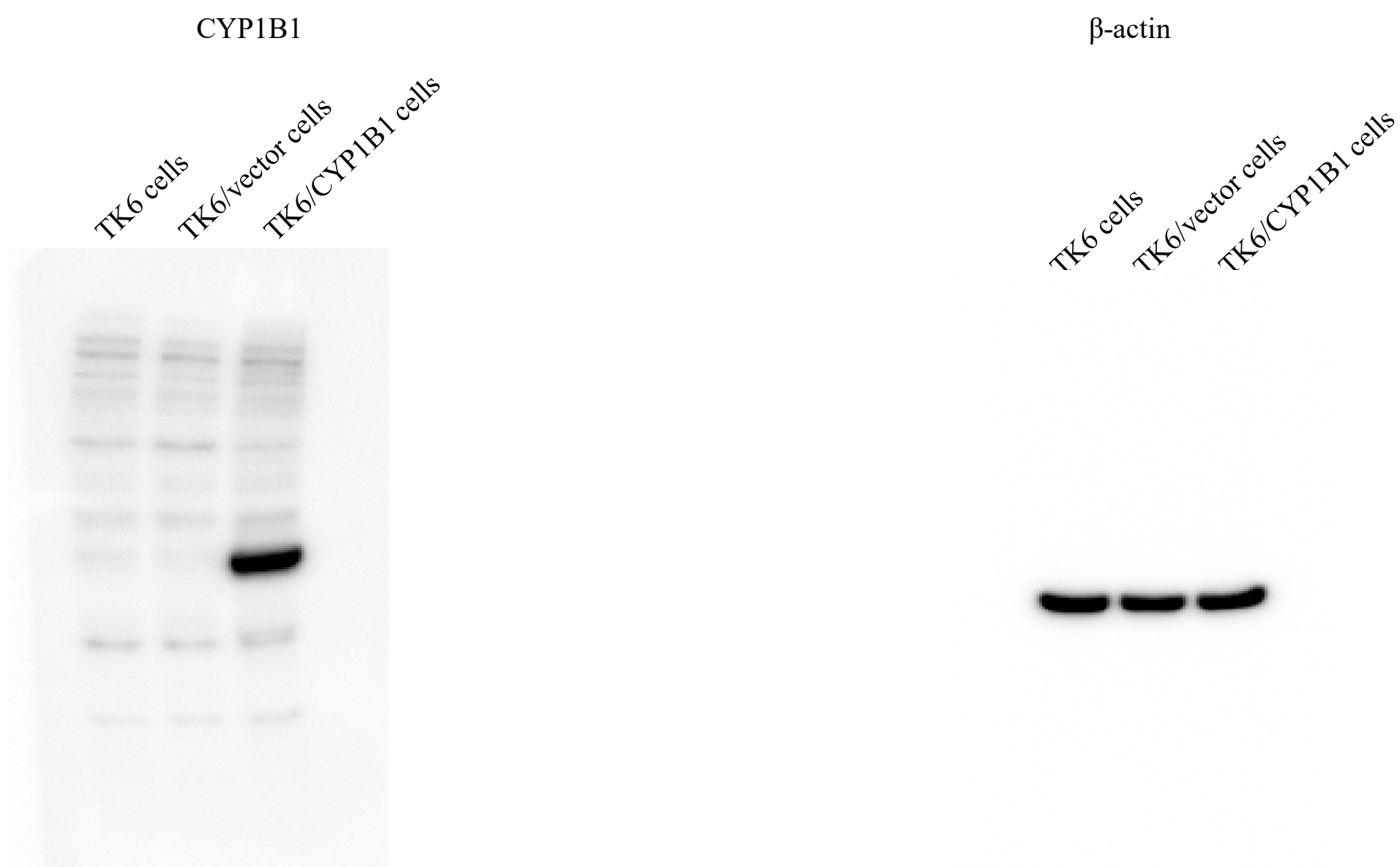

CYP1B1
TK6 cells
TK6/vector cells
TK6/CYP1B1 cells
β-actin
TK6 cells
TK6/vector cells
TK6/CYP1B1 cells

Figure 2

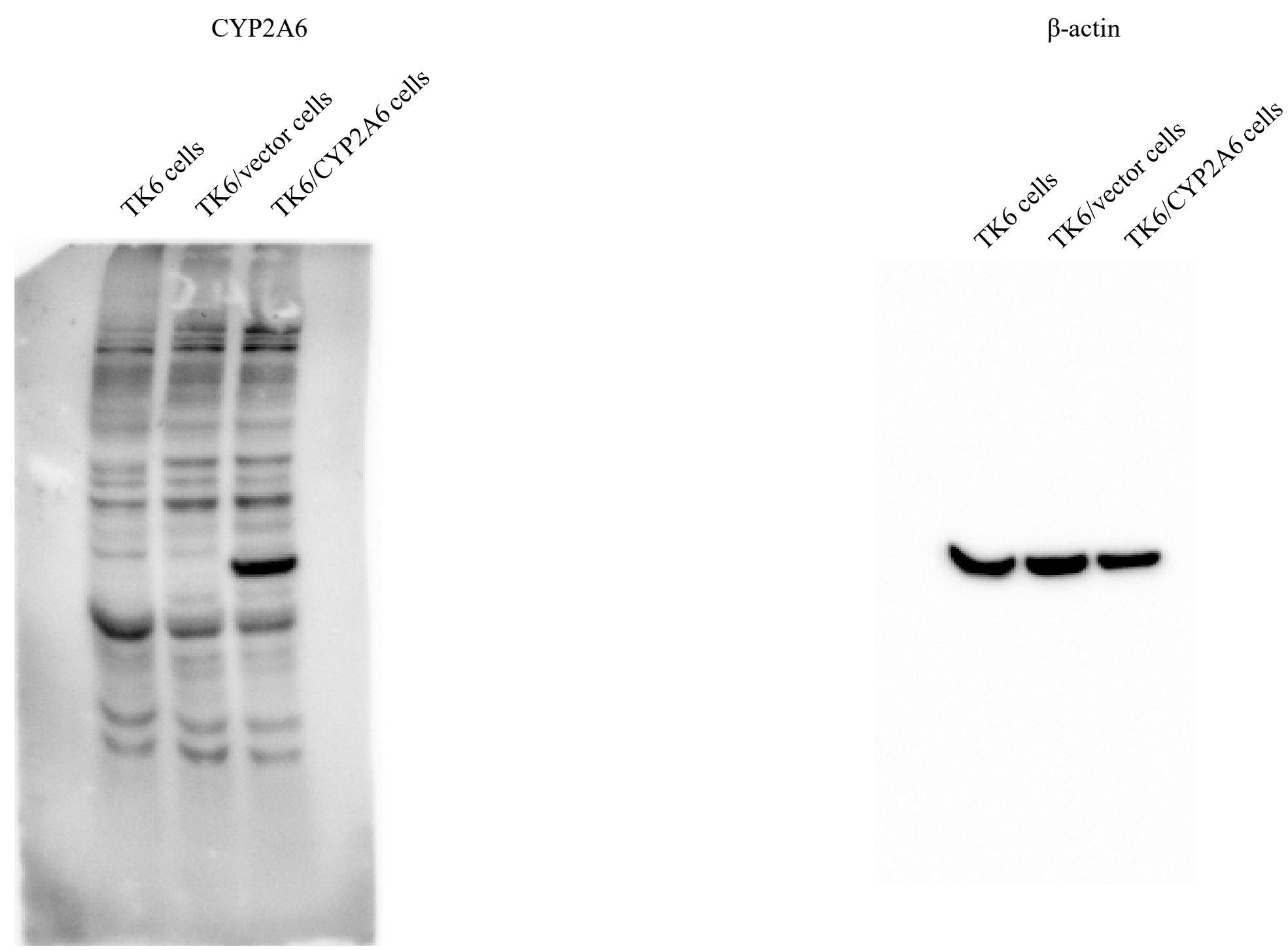
CYP2A6
TK6 cells
TK6/vector cells
TK6/CYP2A6 cells
β-actin
TK6 cells
TK6/vector cells
TK6/CYP2A6 cells

Figure 2

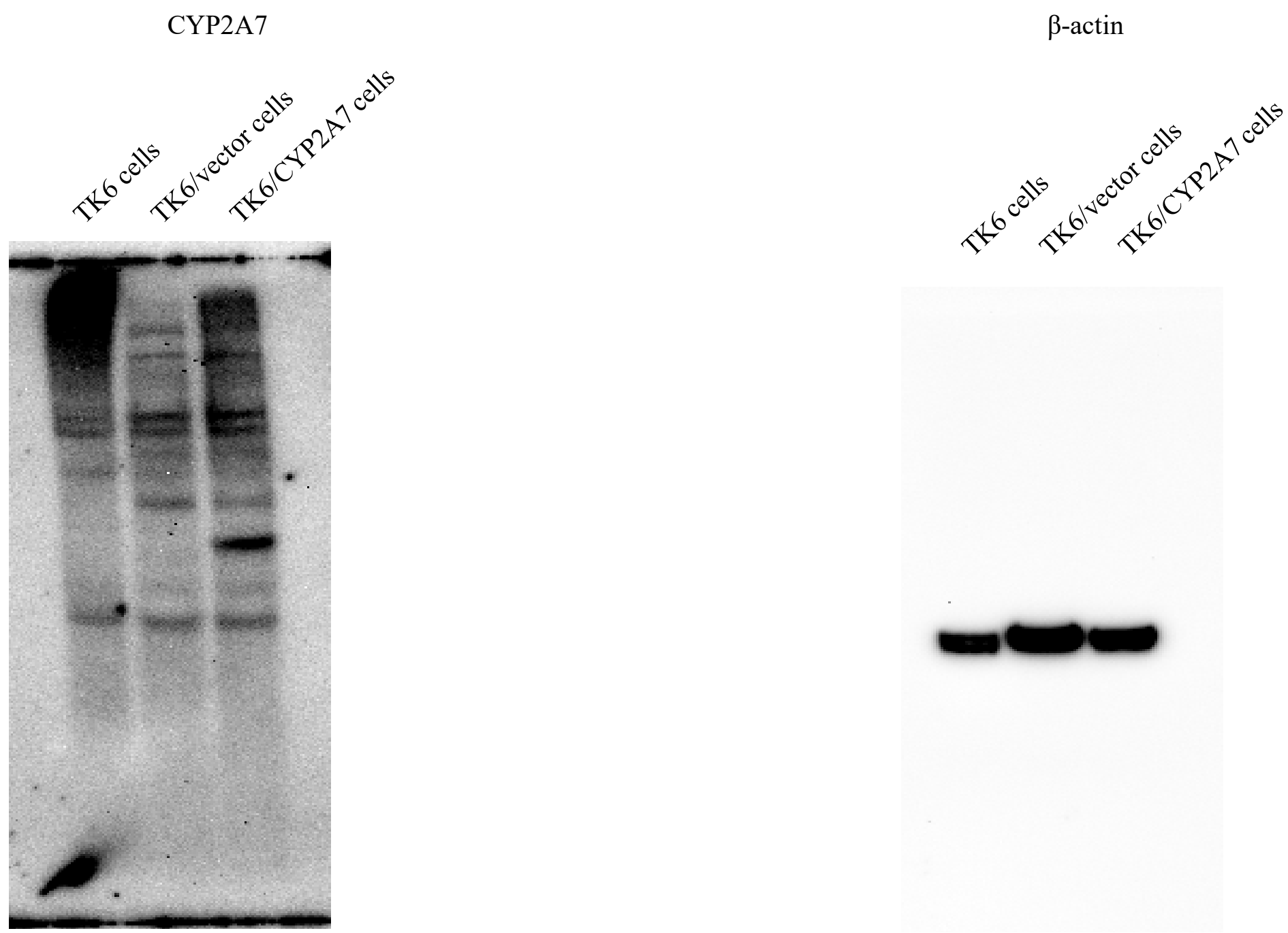

CYP2A7
TK6 cells
TK6/vector cells
TK6/CYP2A7 cells
β-actin
TK6 cells
TK6/vector cells
TK6/CYP2A7 cells

Figure 2

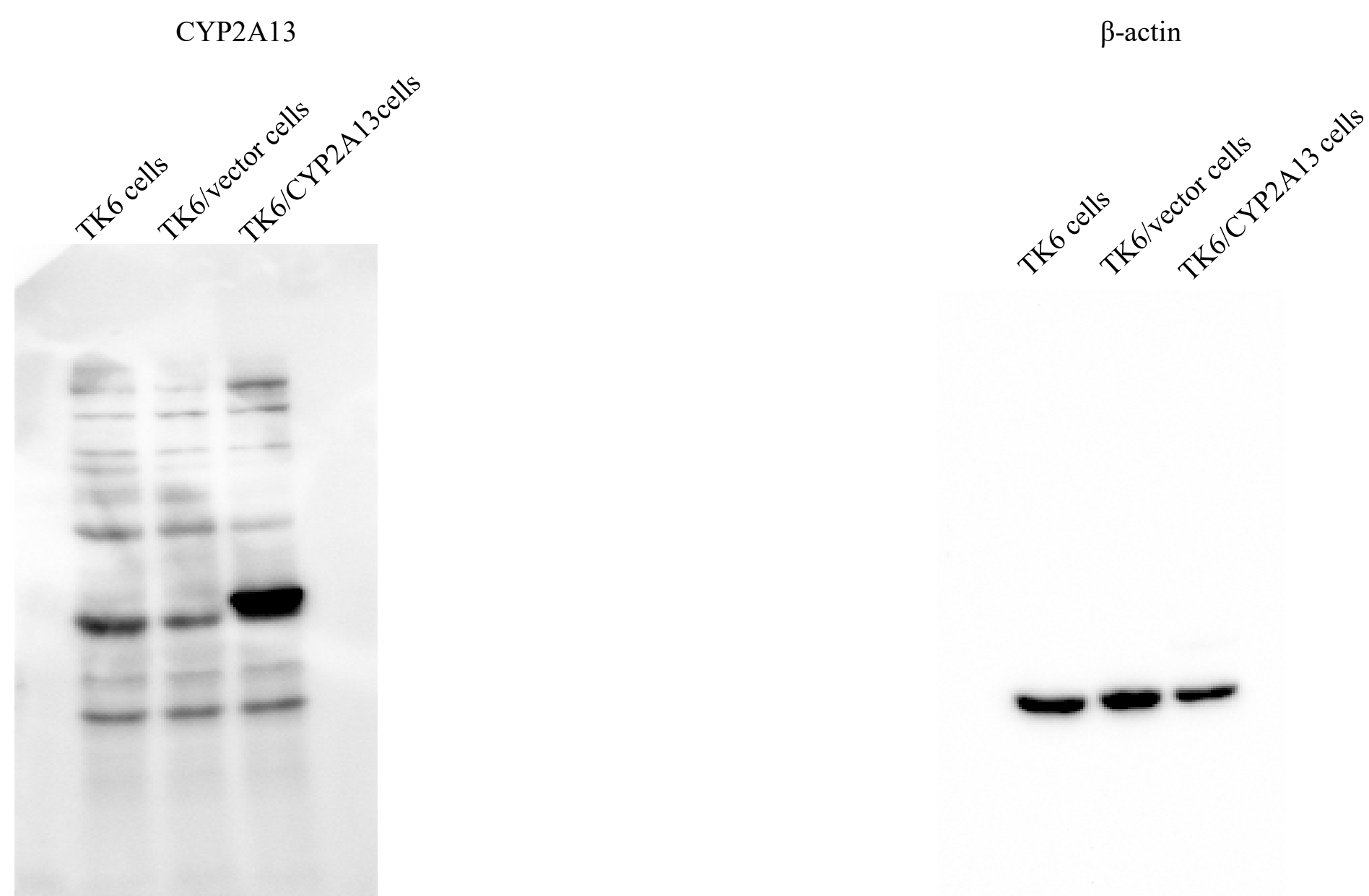
CYP2A13
TK6 cells
TK6/vector cells
TK6/CYP2A13cells
β-actin
TK6 cells
TK6/vector cells
TK6/CYP2A13 cells

Figure 2

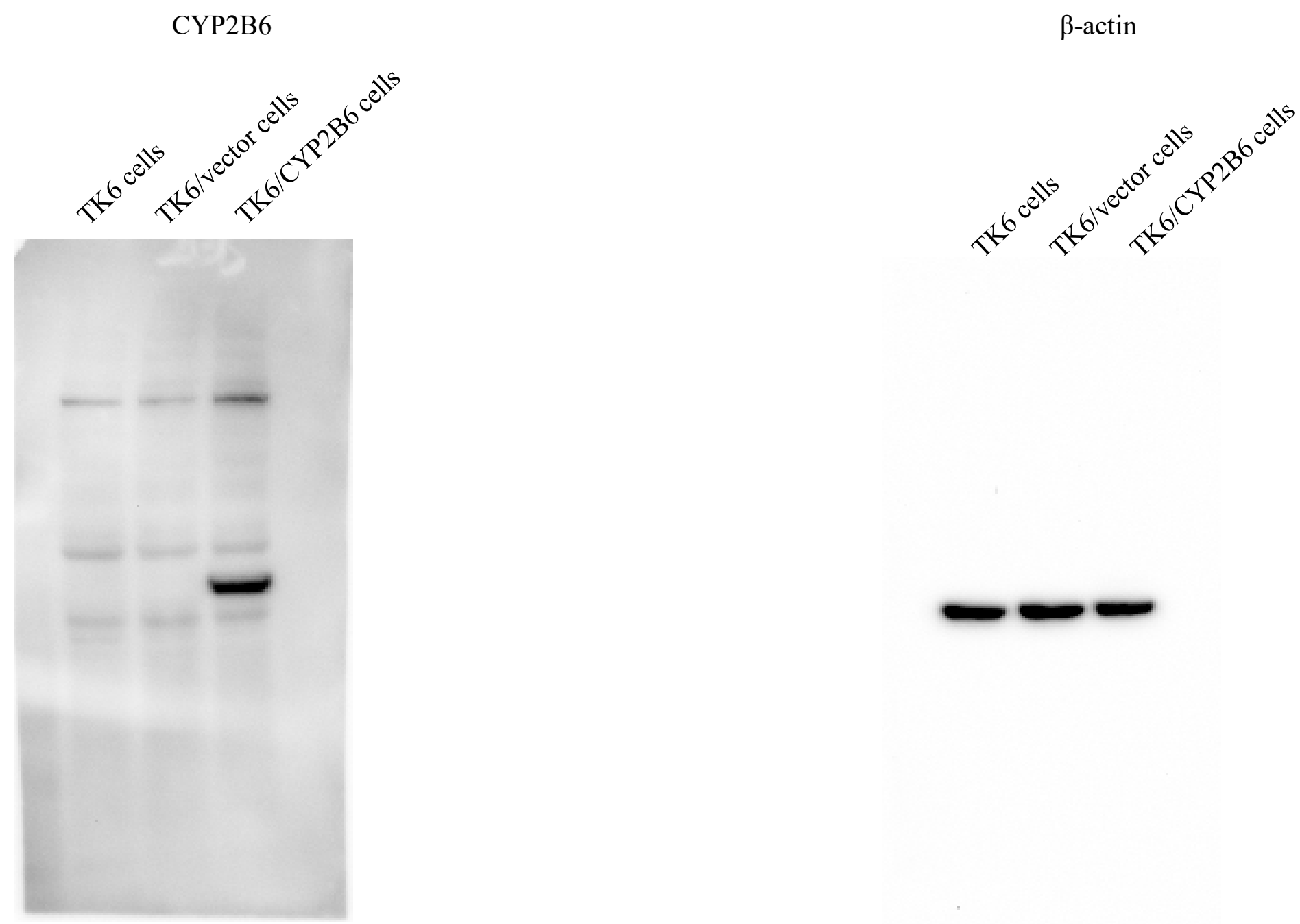
CYP2B6
TK6 cells
TK6/vector cells
TK6/CYP2B6 cells
β-actin
TK6 cells
TK6/vector cells
TK6/CYP2B6 cells

Figure 2

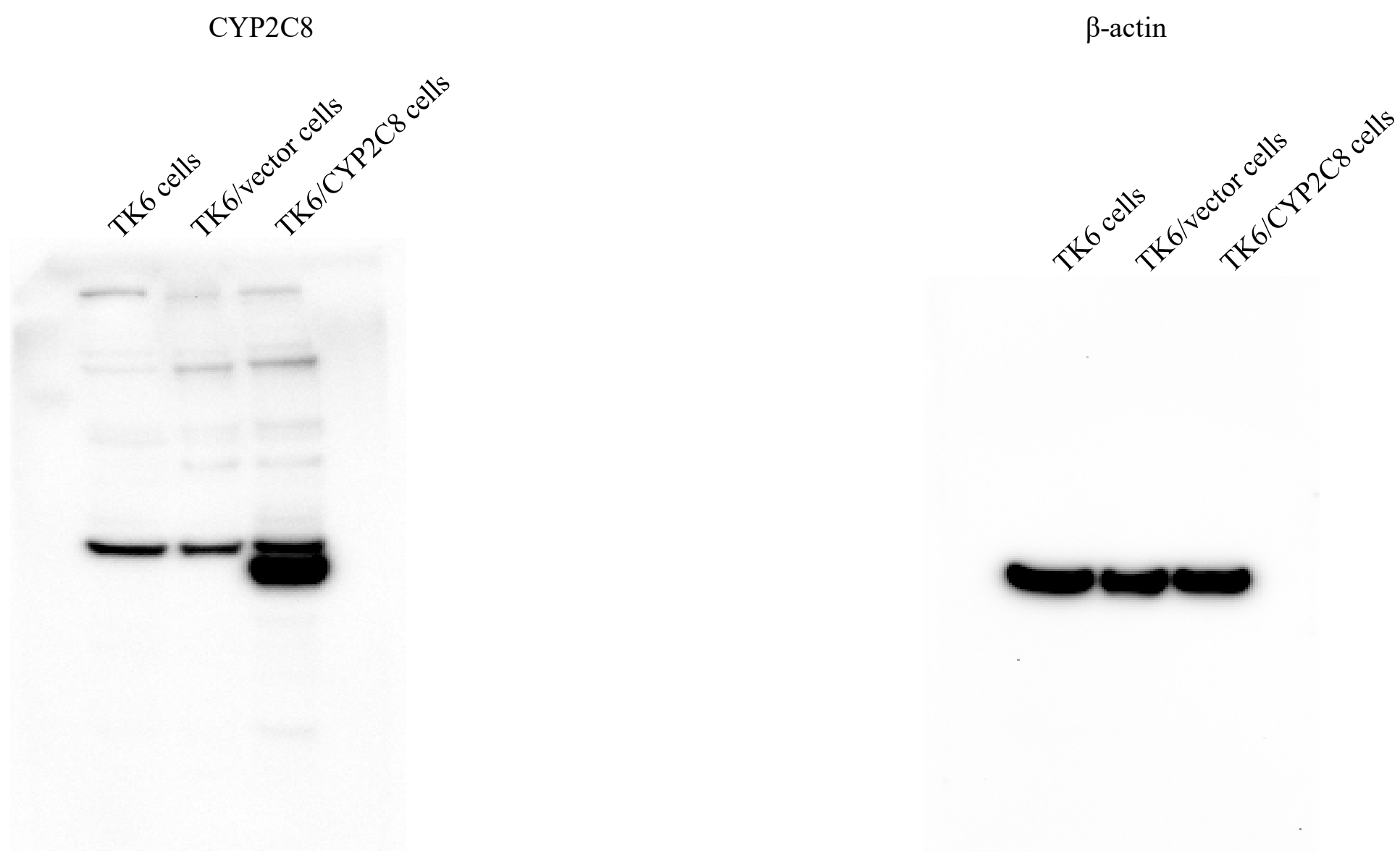
CYP2C8
TK6 cells
TK6/vector cells
TK6/CYP2C8 cells
β-actin
TK6 cells
TK6/vector cells
TK6/CYP2C8 cells

Figure 2

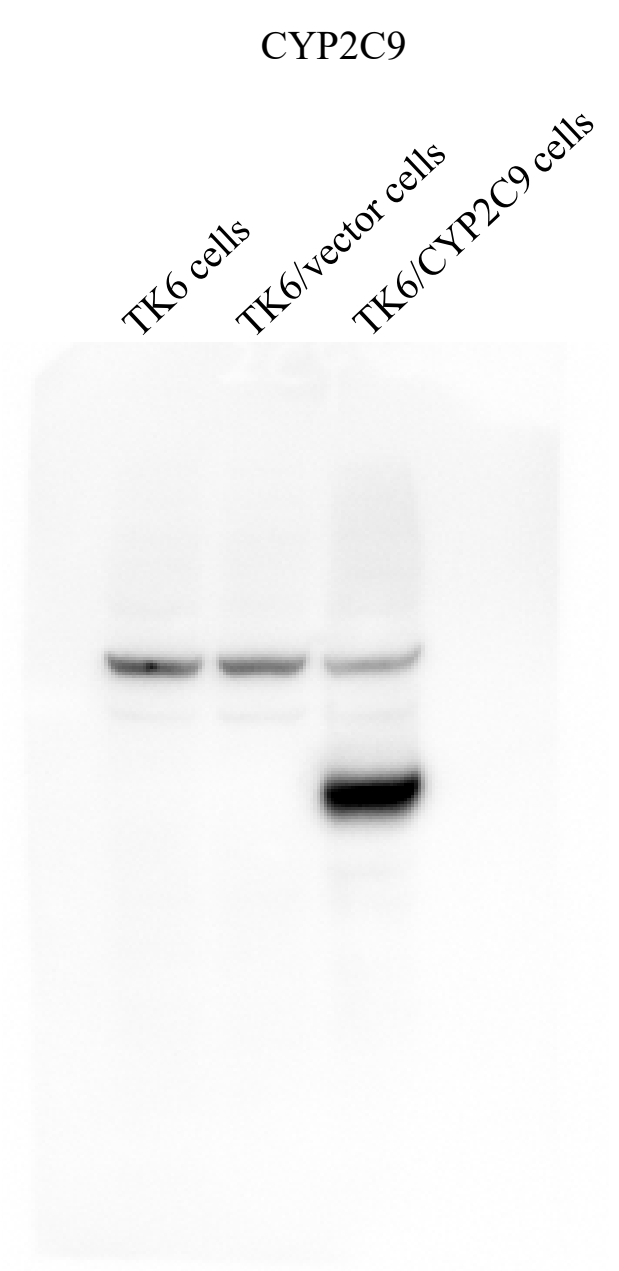
CYP2C9
TK6 cells
TK6/vector cells
TK6/CYP2C9 cells

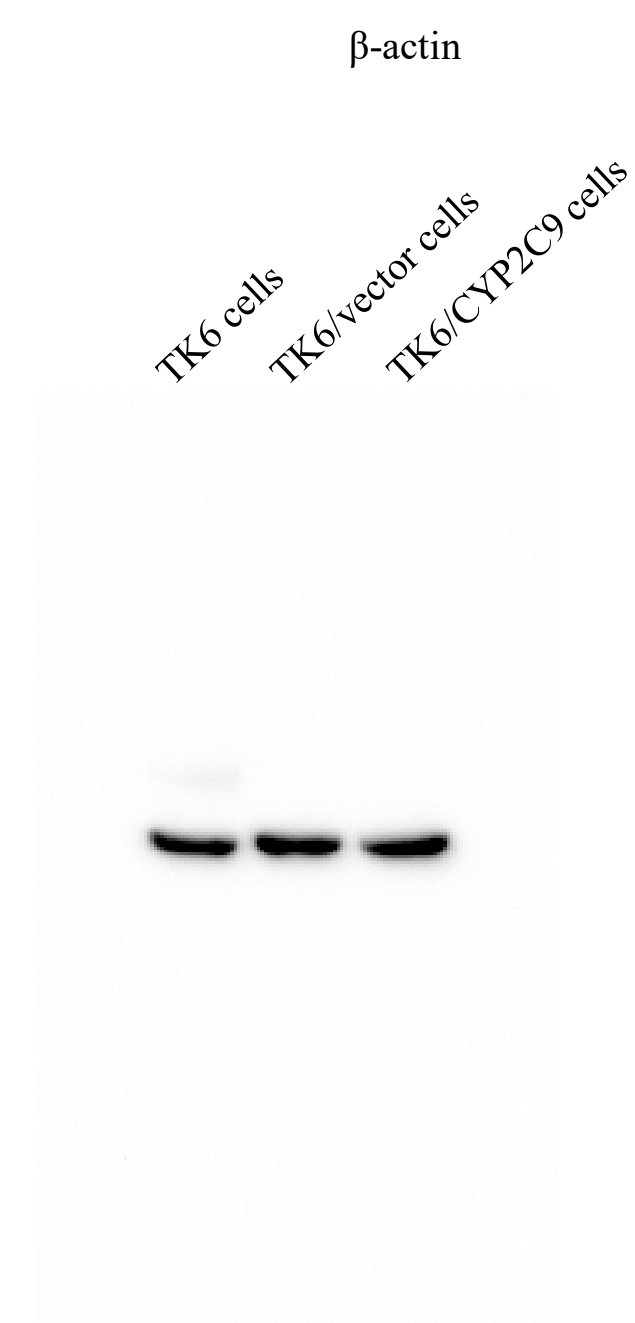
β-actin
TK6 cells
TK6/vector cells
TK6/CYP2C9 cells

Figure 2

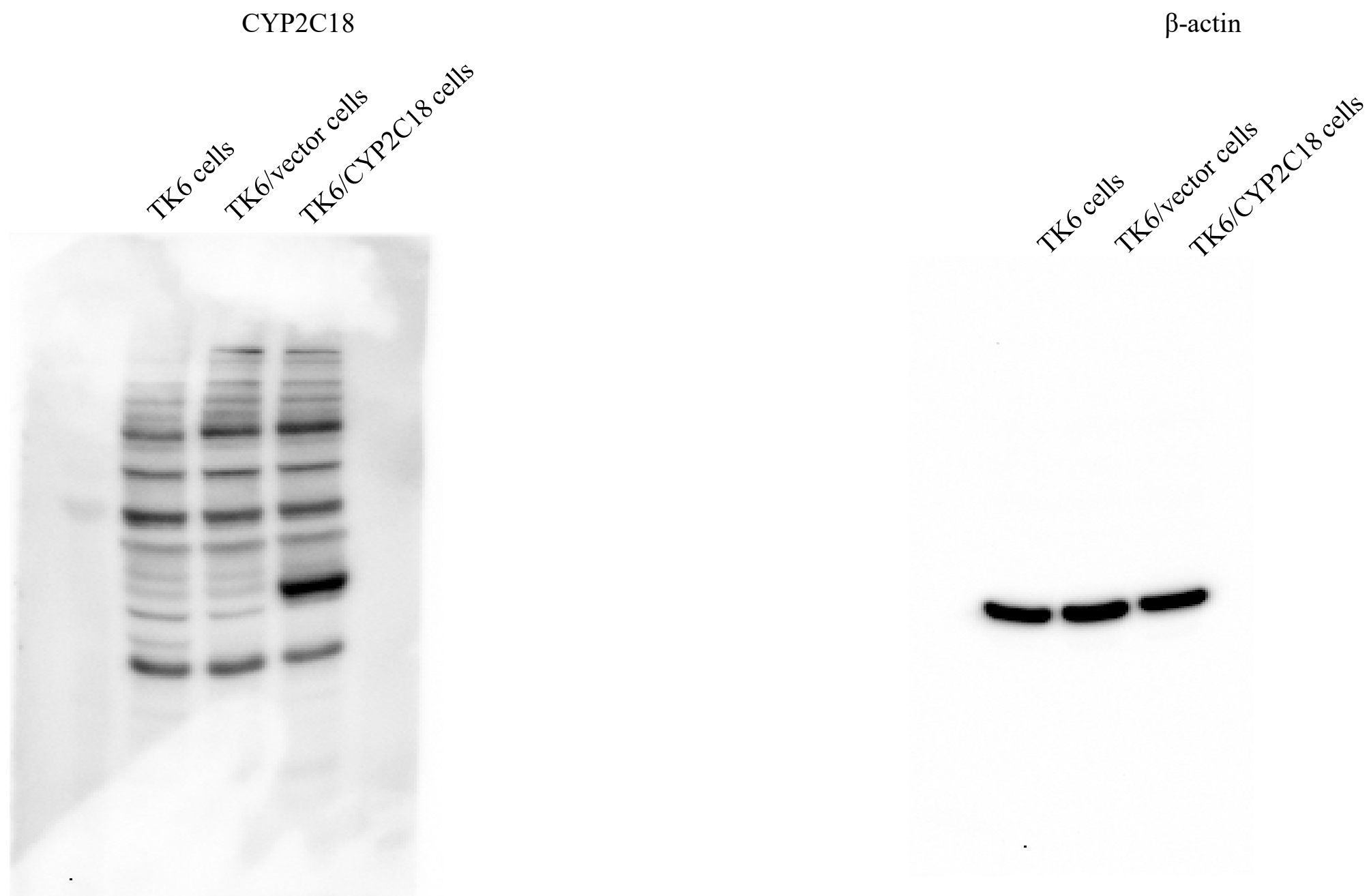

CYP2C18
TK6 cells
TK6/vector cells
TK6/CYP2C18 cells
β-actin
TK6 cells
TK6/vector cells
TK6/CYP2C18 cells

Figure 2

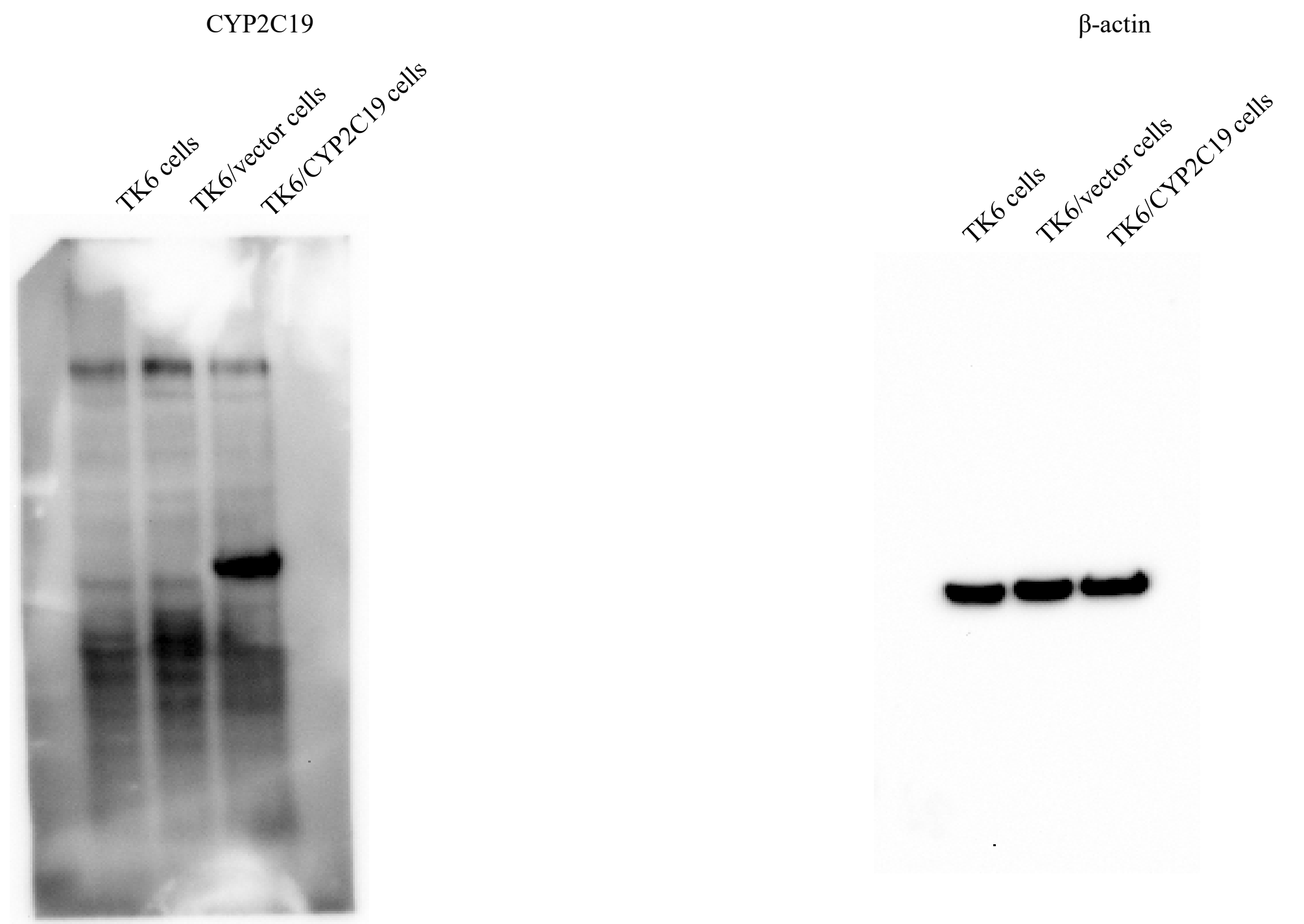
CYP2C19
TK6 cells
TK6/vector cells
TK6/CYP2C19 cells
β-actin
TK6 cells
TK6/vector cells
TK6/CYP2C19 cells

Figure 2

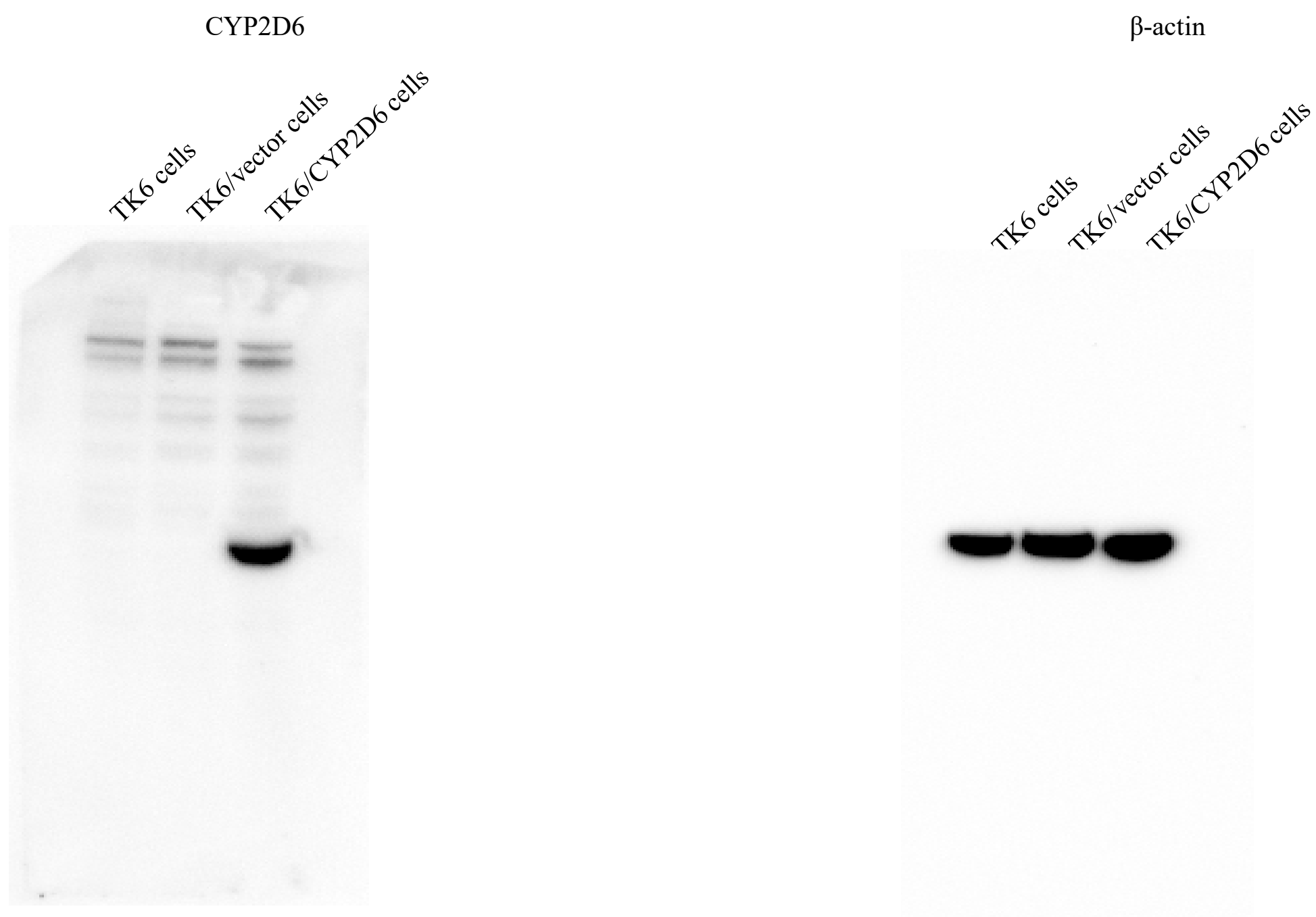
CYP2D6
TK6 cells
TK6/vector cells
TK6/CYP2D6 cells
β-actin
TK6 cells
TK6/vector cells
TK6/CYP2D6 cells

Figure 2

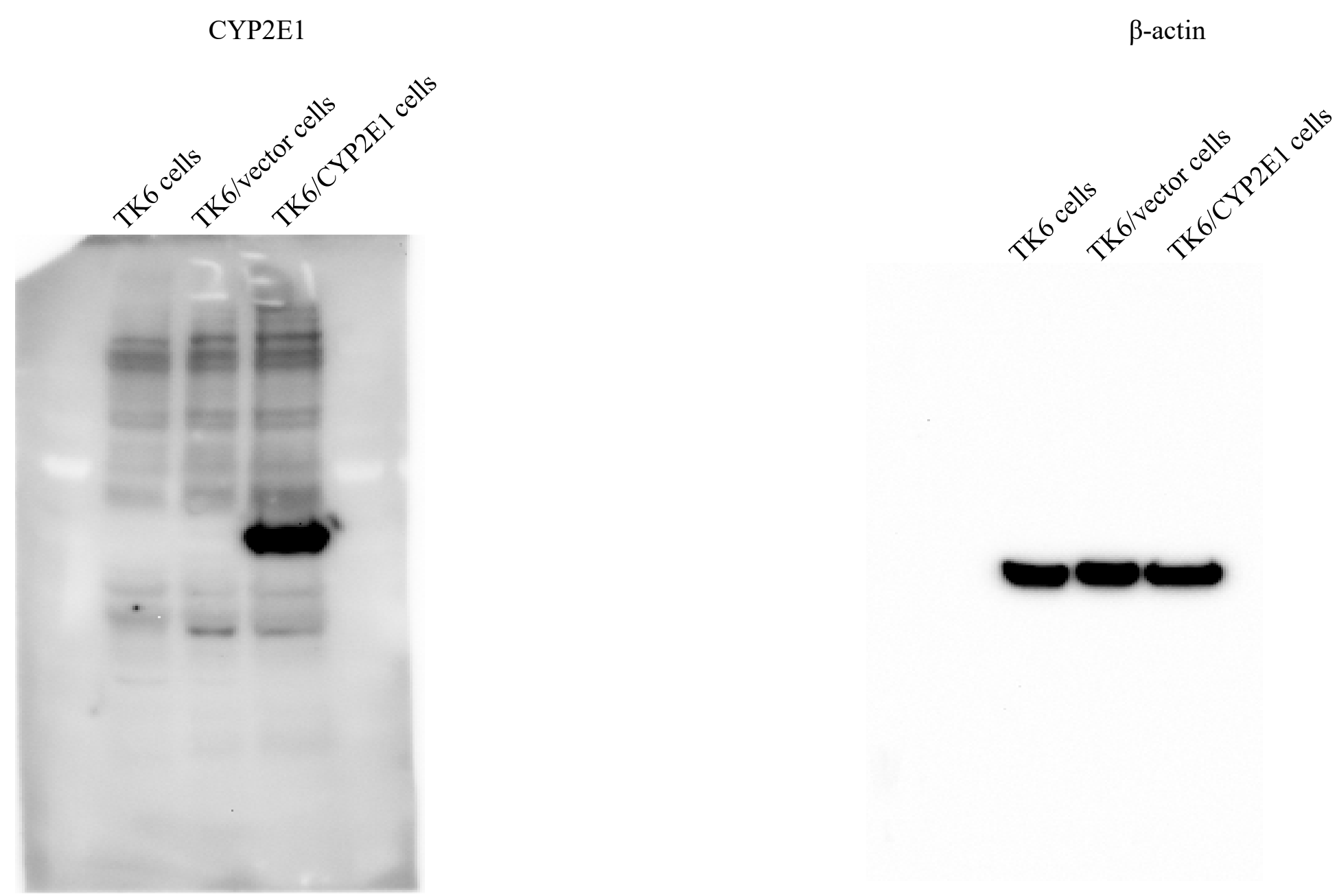
CYP2E1
TK6 cells
TK6/vector cells
TK6/CYP2E1 cells
β-actin
TK6 cells
TK6/vector cells
TK6/CYP2E1 cells

Figure 2

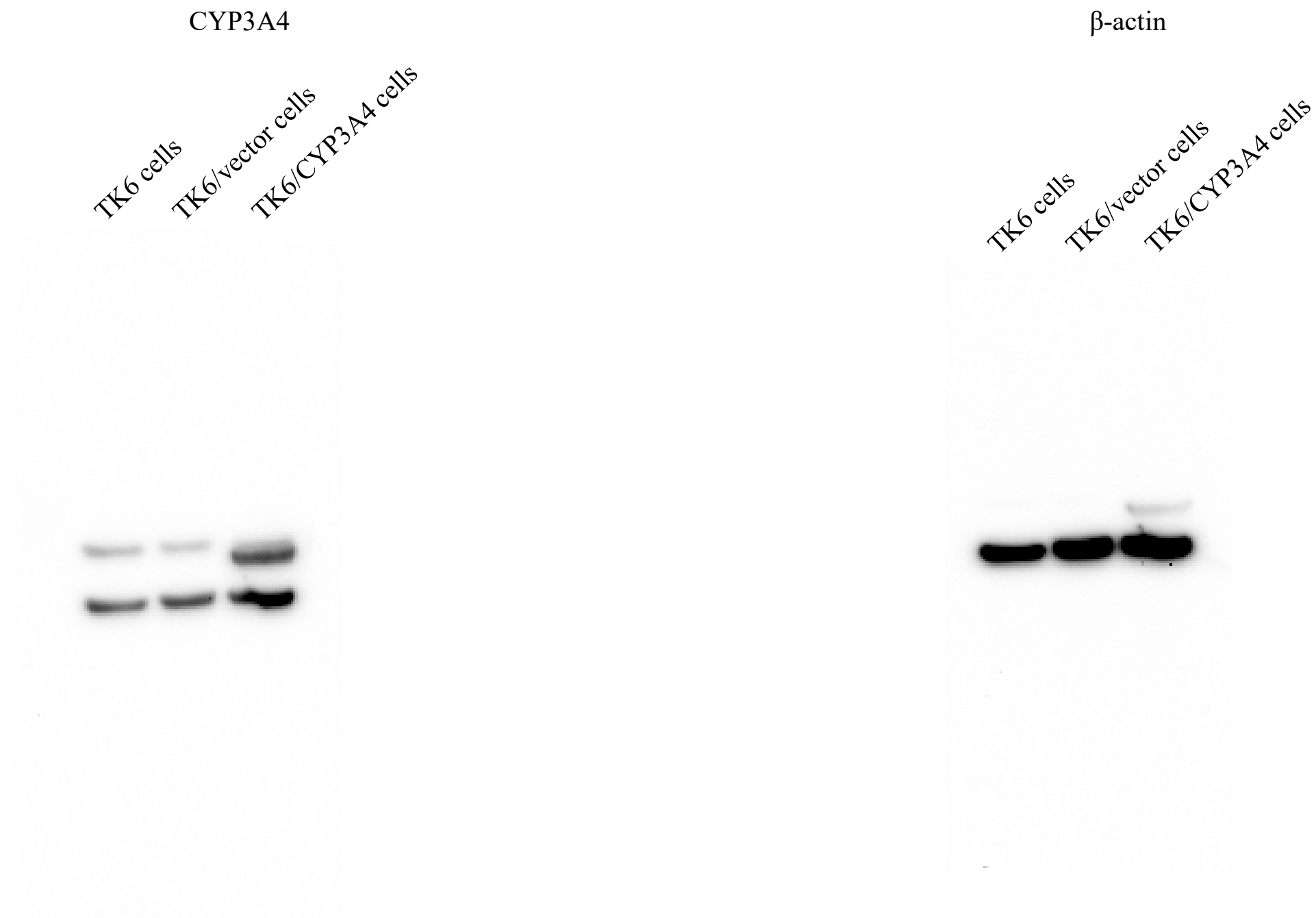
CYP3A4
TK6 cells
TK6/vector cells
TK6/CYP3A4 cells
β-actin
TK6 cells
TK6/vector cells
TK6/CYP3A4 cells

Figure 2

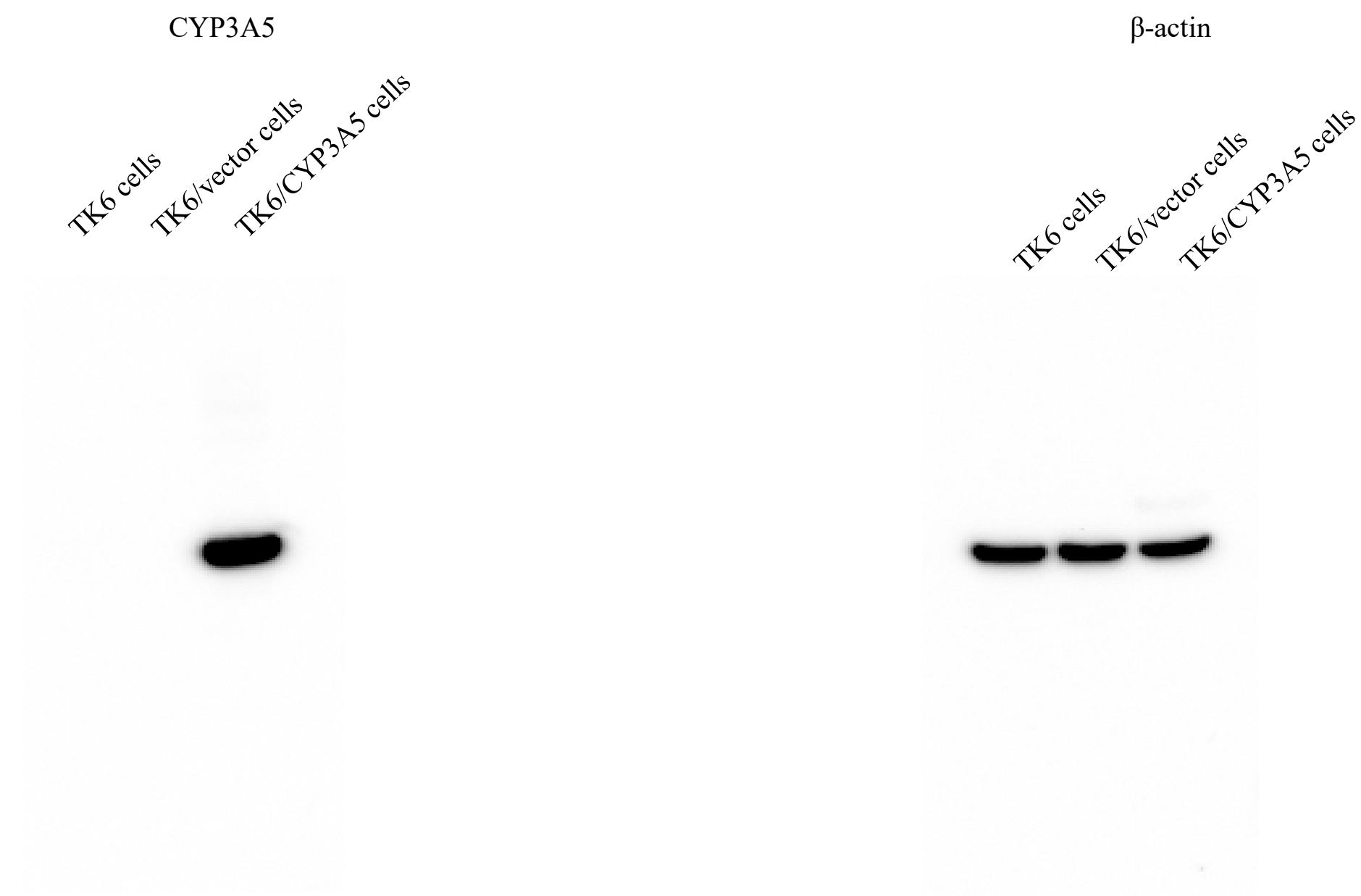
CYP3A5
TK6 cells
TK6/vector cells
TK6/CYP3A5 cells
β-actin
TK6 cells
TK6/vector cells
TK6/CYP3A5 cells

Figure 2

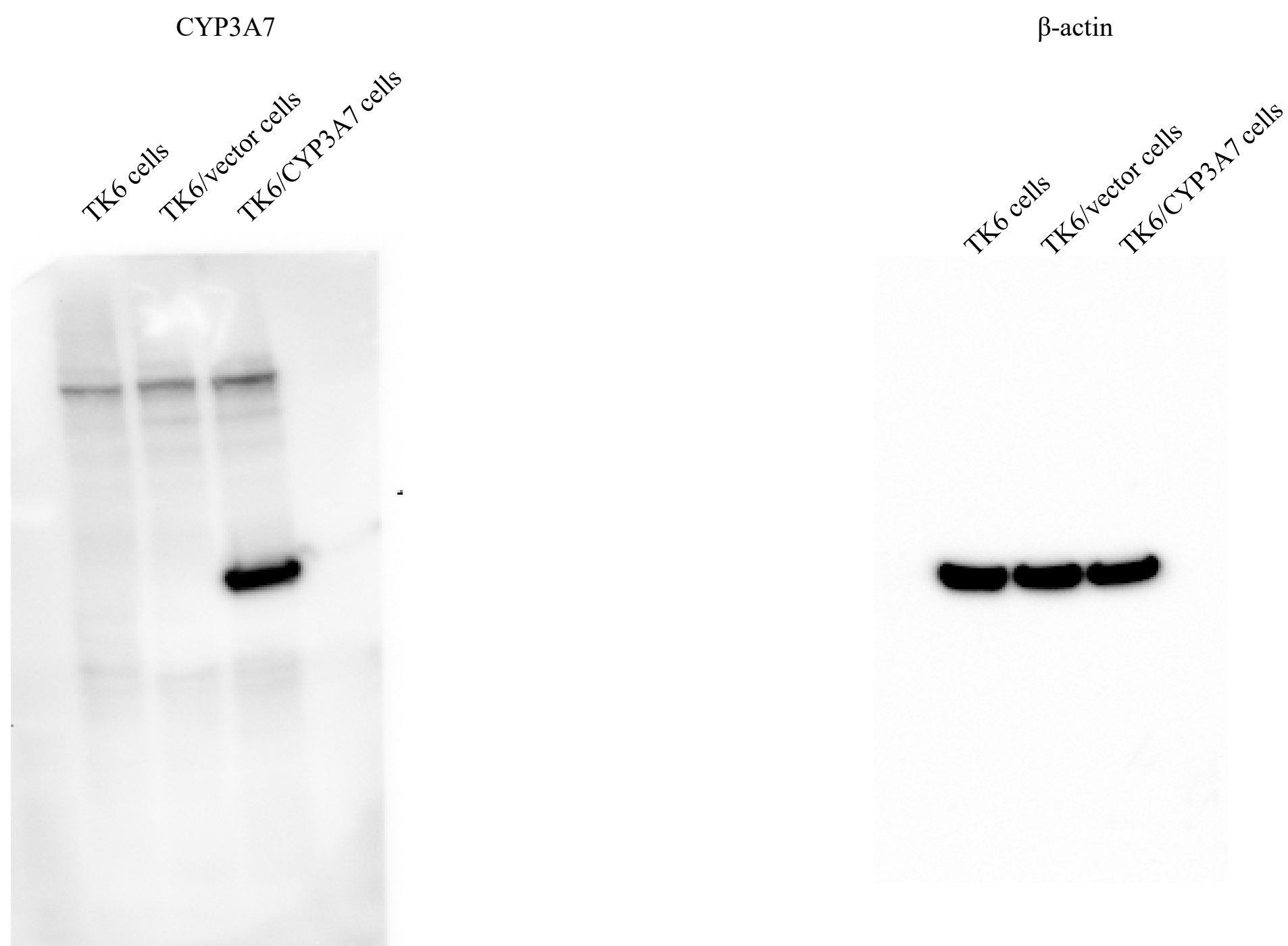
CYP3A7
TK6 cells
TK6/vector cells
TK6/CYP3A7 cells
β-actin
TK6 cells
TK6/vector cells
TK6/CYP3A7 cells

Figure 2

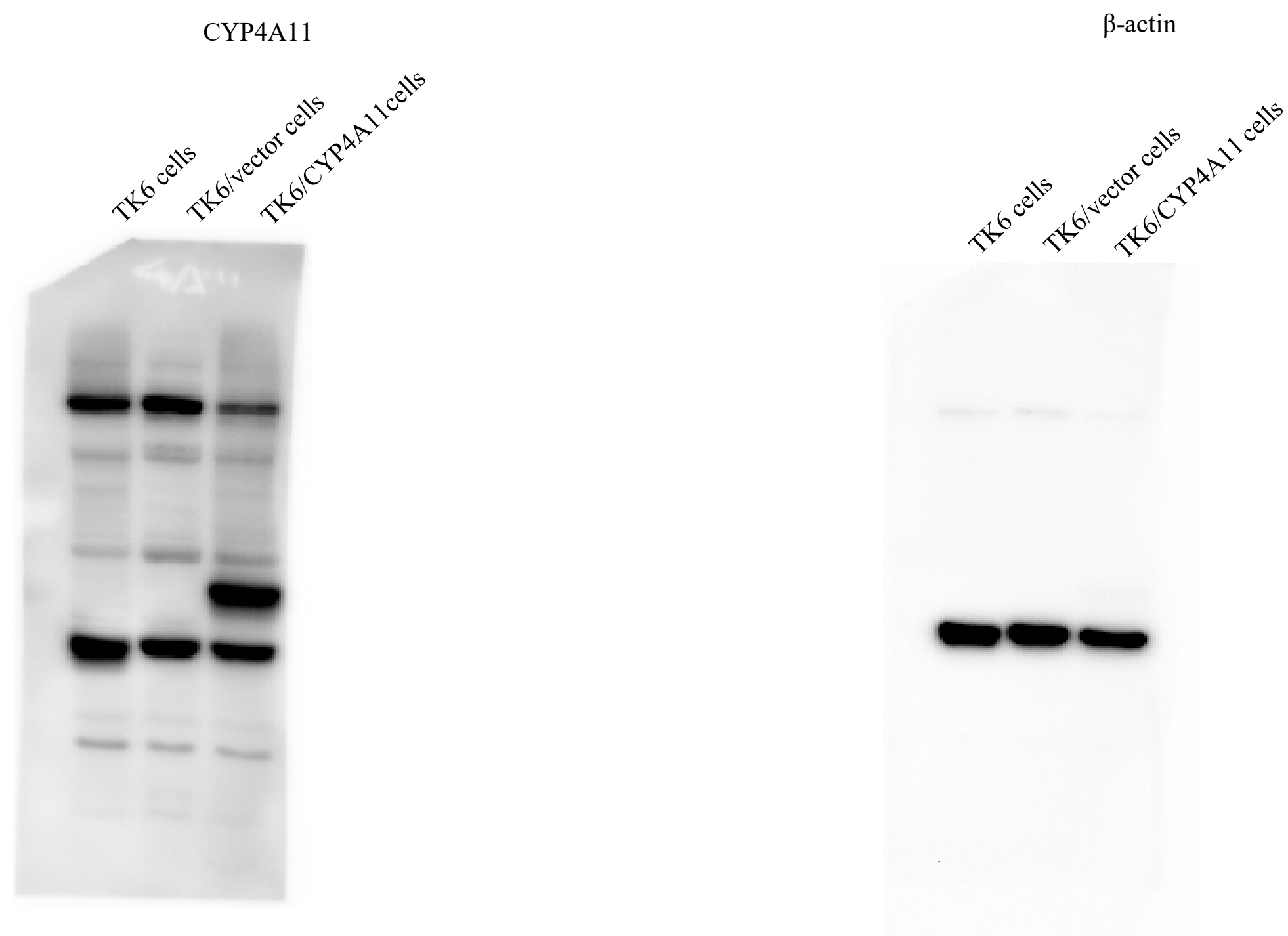
CYP4A11
TK6 cells
TK6/vector cells
TK6/CYP4A11cells
β-actin
TK6 cells
TK6/vector cells
TK6/CYP4A11 cells

Figure 2

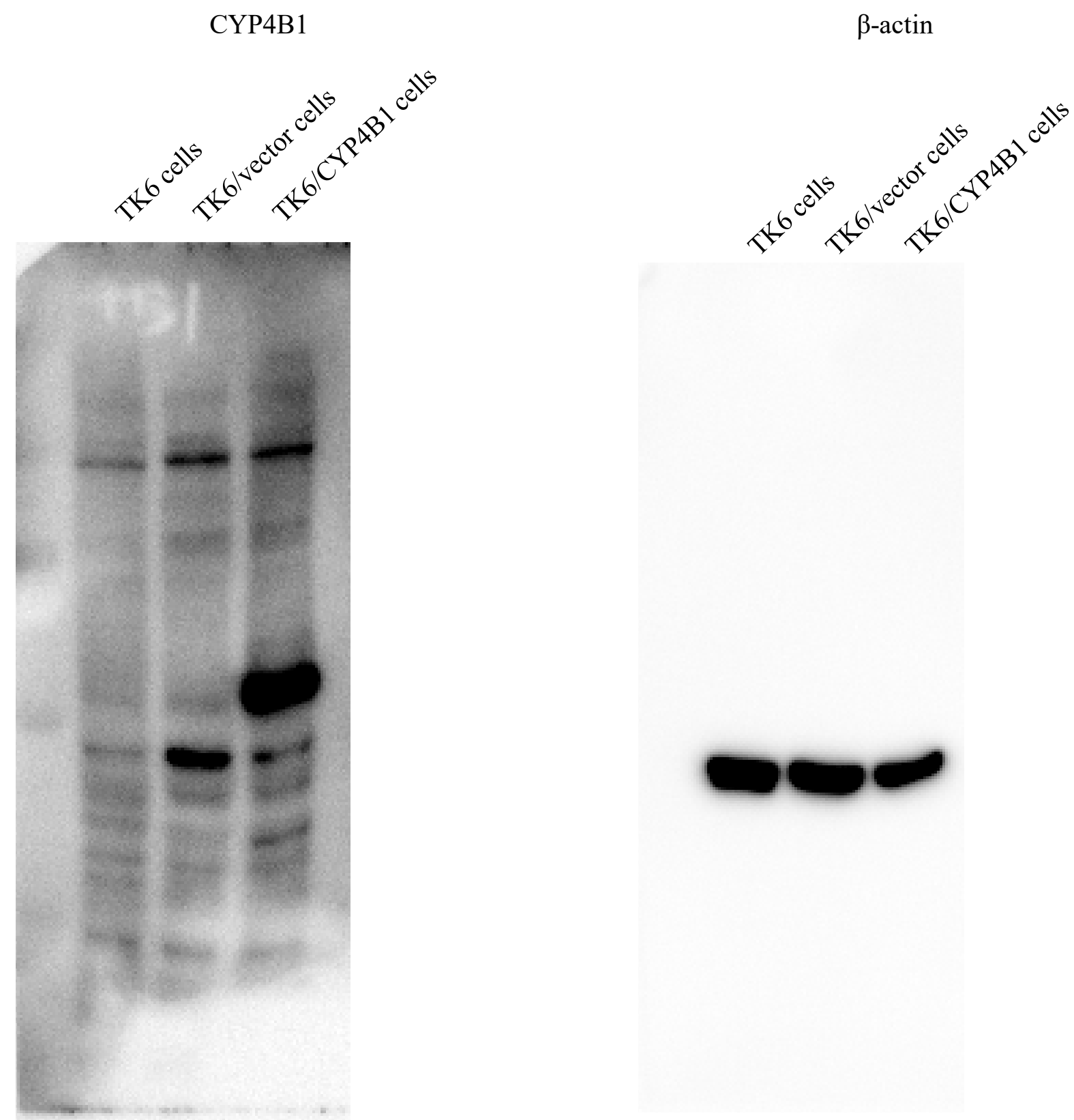

CYP4B1
TK6 cells
TK6/vector cells
TK6/CYP4B1 cells
β-actin
TK6 cells
TK6/vector cells
TK6/CYP4B1 cells

Figure 7A

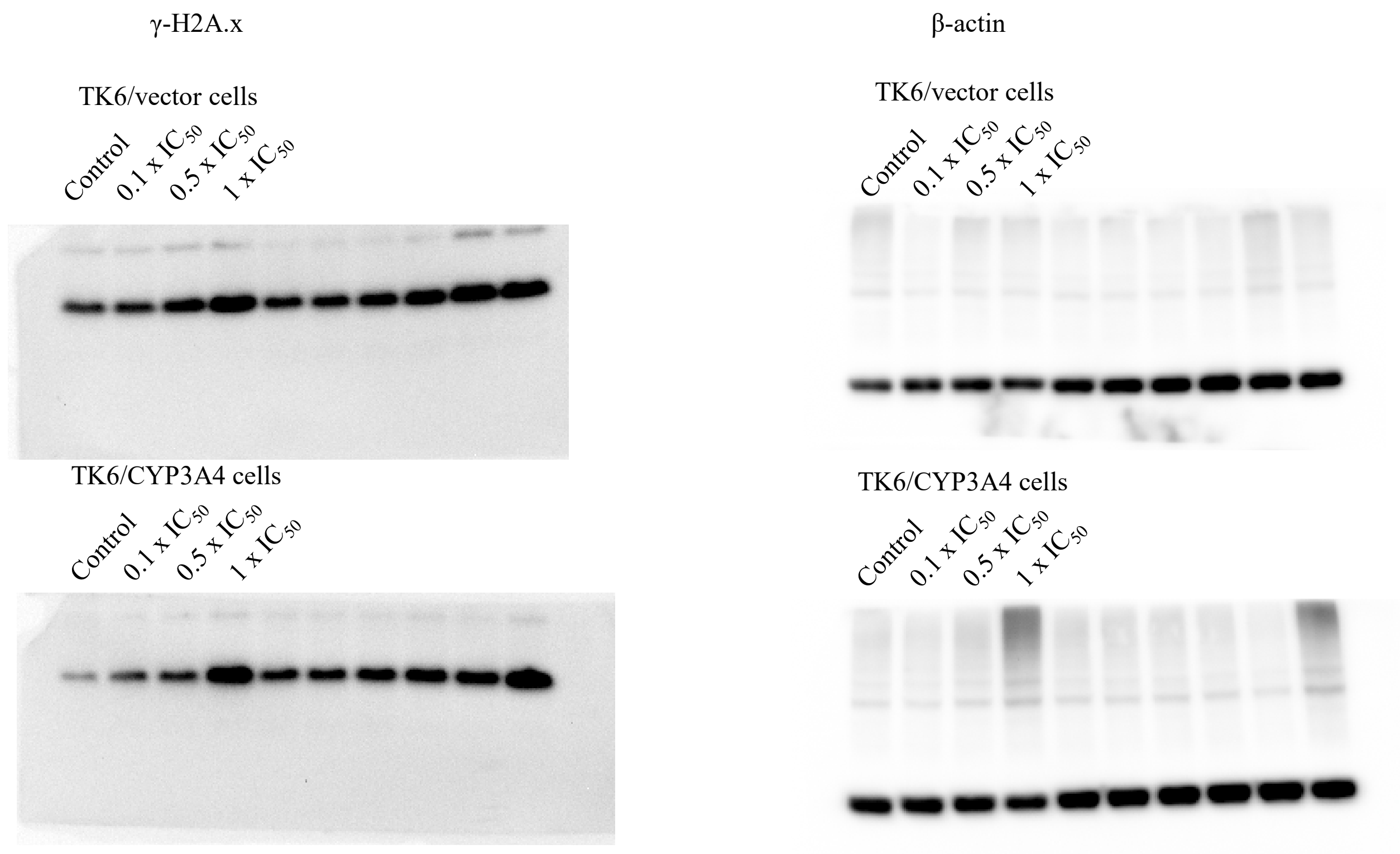
γ-H2A.x
TK6/vector cells
Control
0.1 x IC50
0.5 x IC50
1 x IC50
TK6/CYP3A4 cells
Control
0.1 x IC50
0.5 x IC50
1 x IC50
β-actin
TK6/vector cells
Control
0.1 x IC50
0.5 x IC50
1 x IC50
TK6/CYP3A4 cells
Control
0.1 x IC50
0.5 x IC50
1 x IC50
